# Optimal Threshold Determination for Interpreting Semantic Similarity and Particularity: Application to the Comparison of Gene Sets and Metabolic Pathways Using GO and ChEBI

**DOI:** 10.1371/journal.pone.0133579

**Published:** 2015-07-31

**Authors:** Charles Bettembourg, Christian Diot, Olivier Dameron

**Affiliations:** 1 Université de Rennes 1, Rennes, France; 2 INRA, UMR1348 PEGASE, Saint-Gilles, France; 3 Agrocampus OUEST, UMR1348 PEGASE, Rennes, France; 4 IRISA, Campus de Beaulieu, Rennes, France; 5 INRIA, Rennes, France; Hellas, GREECE

## Abstract

**Background:**

The analysis of gene annotations referencing back to Gene Ontology plays an important role in the interpretation of high-throughput experiments results. This analysis typically involves semantic similarity and particularity measures that quantify the importance of the Gene Ontology annotations. However, there is currently no sound method supporting the interpretation of the similarity and particularity values in order to determine whether two genes are similar or whether one gene has some significant particular function. Interpretation is frequently based either on an implicit threshold, or an arbitrary one (typically 0.5). Here we investigate a method for determining thresholds supporting the interpretation of the results of a semantic comparison.

**Results:**

We propose a method for determining the optimal similarity threshold by minimizing the proportions of false-positive and false-negative similarity matches. We compared the distributions of the similarity values of pairs of similar genes and pairs of non-similar genes. These comparisons were performed separately for all three branches of the Gene Ontology. In all situations, we found overlap between the similar and the non-similar distributions, indicating that some similar genes had a similarity value lower than the similarity value of some non-similar genes. We then extend this method to the semantic particularity measure and to a similarity measure applied to the ChEBI ontology. Thresholds were evaluated over the whole HomoloGene database. For each group of homologous genes, we computed all the similarity and particularity values between pairs of genes. Finally, we focused on the PPAR multigene family to show that the similarity and particularity patterns obtained with our thresholds were better at discriminating orthologs and paralogs than those obtained using default thresholds.

**Conclusion:**

We developed a method for determining optimal semantic similarity and particularity thresholds. We applied this method on the GO and ChEBI ontologies. Qualitative analysis using the thresholds on the PPAR multigene family yielded biologically-relevant patterns.

## Introduction

### Need for thresholds

Comparing several gene sets to identify and quantify the features they share and the features that differentiate them is central to the functional analysis of gene sets [1–3]. These operations hinge on comparing sets of Gene Ontology (GO) terms [4]. The links between genes and GO terms are provided by the Gene Ontology Annotation (GOA) database for multiple species [5]. Numerous semantic similarity measures have been developed [6–8]. We recently proposed to combine semantic similarity measures and a new semantic particularity measure to improve the results of gene set analysis [9]. The analysis of results on similarity and particularity is based on an interpretation that contrasts the genes with particular functions among similar genes. The main focus of studies to date has been on defining the measures, but there is no extensive study on the interpretation of the values obtained with these measures. As a result, interpretation is frequently based on either an implicit threshold (for example: “a similarity of 0.83 is *high enough* to consider that two genes are similar”) or an arbitrary one (typically 0.5 for measures in [0;1] even though no mathematical property of the measures supports this choice). Moreover, the value of these thresholds may vary over time, as both GO and GOA evolve [10]. Here, we propose a method to define suitable thresholds based on analysis of the distributions of similarity values. We then extend this method to the semantic particularity measure and to a similarity measure applied to the Chemical Entities of Biological Interest ontology (ChEBI) [11].

## Metrics background

The GO terms annotating genes describe the biological processes, molecular functions and cellular components each gene is involved in. If these terms were independent, functional gene characterization could be performed by a straightforward set-based approach such as the Jaccard index or Dice’s coefficient. However, GO terms are hierarchically-linked, which means the characterization needs to take into account the underlying ontological structure of the GO annotations [12]. There are several semantic similarity measures that exploit the formal representation of the meaning of the terms by considering the relations between the terms.

### Classification of semantic similarity measures

Pesquita *et al.* classified semantic similarity measures into two categories: node and edge-based measures, with some hybrid measures [6].

Node-based measures assign an Information Content (IC) value to each ontology term, with the least-frequent terms given the highest IC value. This IC concept, borrowed from Shannon’s information theory [13], was used to measure similarities using ontologies [14–16] such as WordNet [17]. Node-based measures consider that the similarity between two terms relies on their most informative common ancestor. These measures developed in linguistics have been applied to GO [18, 19], where the IC of a GO term is inversely proportional to the frequency with which it annotates a gene using the Gene Ontology Annotations (GOA) database [5]. In the context of gene comparisons, IC-based measures carry three main limitations tied to their dependence on a GOA-based corpus. First, it can prove difficult or even impossible to obtain a relevant corpus. GOA provides single and multi-species tables of annotation. Although using a species-specific table is well suited to intra-species comparisons, it becomes problematic for inter-species comparisons. Second, using a multi-species table (like the UniprotKB table) for cross-species studies is biased towards the most extensively annotated species such as humans or mice. Third, the most extensively studied areas of biology have high annotation frequencies and are therefore less informative and see their importance downgraded, whereas the less-studied areas are artificially emphasized [20–22].

Edge-based measures compute a distance between GO terms using the directed graph topology. This distance can be the shortest path between two compared terms [23] or the length of the path between the root of the ontology and the lowest common ancestor of the compared terms [24–28]. This root to ancestor distance makes terms with a deep common ancestor more similar than terms with a common ancestor close to the root. Unlike node-based measures, edge-based measures are not corpus-dependent. However, granularity is not uniform in GO, so terms at the same depth can have different levels of specificity [29].

Hybrid measures combine different aspects of node-based and edge-based measures. Wang *et al.*’s measure assigns each term a “semantic value” that represents how informative the term is, which conforms to the node-based approach [30]. However, the semantic value of a term is obtained by following the path from this term to the root and summing the semantic contributions of all the ancestors of this term. As semantic value depends on ontology topology, it also conforms to the edge-based approach. Most hybrid measures are designed to compare terms but not sets of terms (as needed to compare genes). Common approaches proposed to compare genes consider the average [18], the maximum [31] of all pairwise similarities, or only the best matching pairs [32, 33]. Pesquita *et al.* concluded that best-match average variants are the best overall. They also highlighted a graph-based groupwise approach that avoids combining pairwise similarities between terms. Several measures employ this groupwise approach [34–37], including the simUI and simGIC measures used by Ferreira *et al.* to compute similarities on ChEBI [38]. Pesquita *et al.* do not single out any specific semantic similarity measure as the best, as the optimal measure will depend on the data to compare and the level of detail expected in the results. The main advantage of Wang’s measure over pure node-based measures is that unlike the IC, the semantic value is not GOA-dependent, which thus makes it well suited to cross-species comparisons.

Semantic similarity measures typically focus on what is common between the two compared entities. We recently developed a semantic particularity measure to also take into account what distinguishes each compared entity from the other one [9]. The semantic particularity of a set of GO terms “Sg1” compared to another set of GO terms “Sg2” depends on the informativeness measure of the “Sg1” terms that are not in “Sg2”. This informativeness measure is Wang’s semantic values or an IC value. This particularity concept should be used in combination with semantic similarity in order to improve the functional analysis of gene sets.

Data analysis often hinges on a qualitative interpretation of the similarity values in order to contrast similar and dissimilar pairs of genes. This discretization of the similarity and particularity values makes the interpretation easier. It determines whether a functional difference between two genes is or is not marginal. However, there has never been a systematic analysis of the optimal threshold value separating similar from dissimilar. Some studies avoid the problem by focusing only on “high” or “low” values (without mentioning when a value reaches this point). Other studies draw the line at 0.5 (for no other reason than the fact that 0.5 is the mid-range value of the similarity interval). There are cases where a threshold of 0.5 may be ill-adapted. For example, the similarity value between protein tyrosine kinase 2 (PTK2) and Ubiquitin B (UBB) is 0.502 using Wang’s similarity measure on their Biological Processes (BP) annotations. This value is just above the intuitive mid-interval threshold. These two genes are well annotated, with 73 and 79 distinct BP annotations, repectively. According to Entrez Gene, PTK2 is involved in cell growth and intracellular signal transduction pathways triggered in response to certain neural peptides or cell interactions with the extracellular matrix while UBB is required for ATP-dependent, nonlysosomal intracellular protein degradation of abnormal proteins and normal proteins with rapid turnover. These processes cannot be considered “similar”. Consequently, the 0.502 value of similarity should not lead to consider PTK2 and UBB as similar genes according to the BP they participate in.

The main factors influencing the similarity values are: granularity differences in GO, GO topology differences between BP, MF and CC, quantity and “quality” of gene annotations, GO temporal evolution [10]. There is a need for a systematic study of semantic measure values in order to determine optimal similarity and particularity thresholds for the qualitative part of functional gene set analysis. Note that the method for determining these thresholds should also be applicable to all semantic similarity categories as well on other ontologies outside GO.

Here we propose a generic method to define a threshold. We applied this method to a node-based and a hybrid semantic similarity measure as well as to the corresponding semantic particularity measures. All these measures are able to compare two genes. When comparing more than two genes, the measures have to be applied on each pair of genes. These measures are described below.

### Semantic similarity

Lin developed a widely-used node-based similarity measure that employs the IC concept [15]. Several of the tools available have implemented this measure. The IC of a term t depends on its log probability *P*(*t*). Working with GO terms, this IC is inversely proportional to the frequency with which the terms annotate a gene using the Gene Ontology Annotations (GOA) database. When comparing two GO terms t1 and t2 having a most informative common ancestor t0, Lin defines their similarity as follows:

$$Sim\left(t1,t2\right)=\frac{2\times logP(t0)}{logP(t1)+logP(t2)}$$

Wang’s hybrid measure depends solely on GO graph and does not need an annotation corpus, thus allowing cross-species comparisons [30]. For each term, the first step of the measure is to compute the semantic contributions of its ancestors, following:

$$\left\{ \begin{array}{l} S_{A}\left(A\right)=1\\ S_{A}\left(t\right)=max\{w_{e}*S_{A}\left(t^{\prime }\right)\;|\;t^{\prime }\in children\;of\;\left(t\right)\}\;if\;t\neq A \end{array}\right.$$

where S_*A*_(t) is the semantic contribution of term t to term A and w_*e*_ is the semantic contribution factor for edge e linking term t to its child term t’. Following Wang, we used a semantic contribution factor of 0.8 for the “is a” relations and 0.6 for the “part of” relations, and we added a 0.7 factor for the “[positively] [negatively] regulates” relations. Then, for each target term to compare, the semantic value (SV) is the sum of the semantic contributions of all its ancestors:

$$SV\left(A\right)=\sum_{t\in T_{A}}S_{A}\left(t\right)$$

The comparison of two terms A and B is computed as follows:

$$S_{GO}\left(A,B\right)=\frac{\sum_{t\in T_{A}\cap T_{B}}\left(S_{A}\left(t\right)+S_{B}\left(t\right)\right)}{SV(A)+SV(B)}$$

The similarity between a GO term “go” and a set of GO terms “Sg” is:

$$Sim\left(go,Sg\right)=\max_{1⩽i⩽k}\left(S_{GO}\left(go,go_{i}\right)\right)$$

Finally, the similarity between two genes G1 and G2 is:

$$Sim\left(G1,G2\right)=\frac{\sum_{1\leq i\leq m}\left(Sim\left(go_{1i},Sg2\right)\right)+\sum_{1\leq j\leq n}\left(Sim\left(go_{2j},Sg1\right)\right)}{m+n}$$

Gentleman developed a graph-based measure for the R package GOstats called simUI [36]. simUI defines the semantic similarity between two sets of terms corresponding to two sub-graphs of the ontology as the ratio of the number of terms in the intersection of those graphs to the number of GO terms in their union.

Pesquita *et al.* proposed simGIC, a method combining the graph-based simUI metric with the IC of the terms involved in the computation [37]. In simGIC, each term is weighted by its IC.

### Semantic particularity

In a previous article, we defined the semantic particularity of a set of GO terms Sg1 compared to another set of GO terms Sg2 [9].

Some of the terms of Sg1 that are not members of Sg2 may be linked in the graph. Taking several linked terms into account would result in considering them several times over. To overcome this issue, the particularity measure focuses only on those terms of Sg1 that do not have any descendant in Sg1 and that are not members of Sg2. Some of these terms might be ancestors of terms of Sg2 and should be considered common to Sg1 and Sg2. Sg* is the union of Sg and the sets of ancestors of each term of Sg. MPT(Sg1, Sg2) is the set of the most particular terms of Sg1 compared to Sg2, i.e. the set of terms of Sg1 that do not have any descendant in Sg1 and that are not members of Sg2*. PI(Sg1, Sg2) is the particular informativeness (PI) of a set of GO terms Sg1 compared to another set of GO terms Sg2, i.e. the sum of the differences between the informativeness (I) of each term *t*_*p*_ of MPT(Sg1, Sg2) and the informativeness of the most informative common ancestor (MICA) between *t*_*p*_ and Sg2. The informativeness measure can be a Wang’s semantic value or an IC value. The PI of a set of terms is the information that is not shared with the other set.

$$PI\left(Sg1,Sg2\right)=\sum_{t_{p}\in MPT\left(Sg1,Sg2\right)}I\left(t_{p}\right)-I\left(MICA\left(t_{p},Sg2\right)\right)$$

PI is normalized to compute Par(Sg1, Sg2), the semantic particularity of the set of GO terms Sg1 compared to the set of GO terms Sg2. MCT(Sg1, Sg2) is the set of the most informative common terms of Sg1 and Sg2, i.e. the set of the terms belonging to the intersection of Sg1* and Sg2* that do not have any descendant in either Sg1* or Sg2*. Par(Sg1, Sg2) is the ratio of PI(Sg1, Sg2) and the sum of the informativeness of most informative Sg1 terms (i.e. those that are Sg1-specific and those that are common with Sg2; the MICA in the PI formula for Sg1-specific terms guarantees that the informativeness of common terms is not counted twice).

$$Par\left(Sg1,Sg2\right)=\frac{PI(Sg1,Sg2)}{PI\left(Sg1,Sg2\right)+\sum_{t_{c}\in MCT\left(Sg1,Sg2\right)}I\left(t_{c}\right)}$$

## Method

We first describe our generic method for determining the optimal threshold for a semantic similarity measure. We then used it on GO for a node-based measure and for a hybrid measure. Finally, we generalize our approach by applying the method to another semantic measure of particularity and to another ontology.

### Similarity threshold determination process

**Fig 1:**
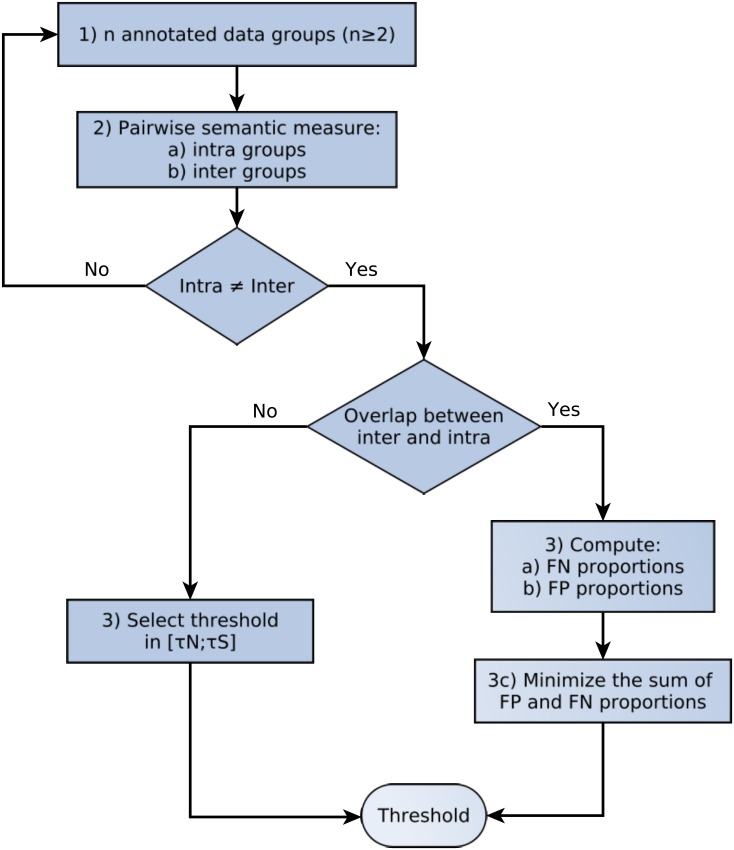
Flowchart for threshold determination. 1) Define at least two distinct groups of genes expected to be similar. 2) Compute the intra- and inter-group similarities and compile the results into S and N distributions. If these two distributions are significantly different, the groups of genes are relevant. 3) If S and N do not overlap, define threshold *τ*_*sim*_ using any value between *τ*_*S*_ (the lowest value of S) and *τ*_*N*_ (the highest value of N). Else, considering every value under the threshold as FN and every value above the threshold as FP, compute the FN proportion in the S distribution (3a) and the FP proportion in the N distribution (3b) for all samples of the similarity threshold between *τ*_*N*_ to *τ*_*S*_. 3c) For each possible threshold value, sum the FN and FP proportions obtained in steps 3a and 3b. The similarity threshold *τ*_*sim*_ is the one that minimizes this sum.

Fig 1 illustrates the process for determining a similarity threshold. This process is composed of three steps:

1. Define at least two different groups of genes for species of interest. Within a group, the genes should share some common characteristics. Genes from different groups should share as few characteristics as possible.
2.
  - In each group, compute the similarities between each pair of genes (i.e. the intra-group similarities). Gather all the similarity results to obtain an S distribution of similar genes.
  - Compute the similarities between each combination of a gene from the first group and a gene from a second group (i.e. the inter-group similarities). Gather all the similarity results to obtain an N distribution of non-similar genes.
3. If the S and N distributions have no overlap between the ranges (min, max), define the threshold *τ*_*sim*_ using any value between *τ*_*S*_ (the lowest value of S) and *τ*_*N*_ (the highest value of N). Else, there are some false negatives (FN) and some false positives (FP):
  - Compute the proportion of FN in the S distribution for all samples of the similarity threshold between *τ*_*N*_ to *τ*_*S*_. In this step, consider every value under the similarity threshold as a FN.
  - Compute the proportion of FP in the N distribution for all samples of the similarity threshold between *τ*_*N*_ to *τ*_*S*_. In this step, consider every value above the similarity threshold as a FP.
  - For each possible threshold value, sum the FN and FP proportions obtained in steps 3a and 3b. The similarity threshold *τ*_*sim*_ is the threshold that minimizes this sum.

We ran a statistical test to determine whether the S and N distributions obtained at step 2 are significantly different. As we cannot consider that the S and N variances are similar, we used an unequal variance t-test (Welch’s t-test) which is the recommended test when considering different-sized distributions like S and N. Welch’s t-test performs better than Student’s t-test when the variances are unequal yet still performs on a par with the Student’s t-test when the variances are equal [39]. If the test concludes that the S and N distributions are non significantly different, the process has to be restarted at its first step.

The minimization at step 3c has to be done on FN and FP proportions as the N and S distributions have different sizes.

We applied this method to compute Lin’s and Wang’s semantic similarity thresholds on GO, the corresponding IC-based and SV-based semantic particularity thresholds on GO, and the simUI and simGIC thresholds on ChEBI. For all the pairs of genes compared, we used the GO annotations from the August 2013 version of GOA. We computed Lin’s similarity with the GOSemSim R package [40] (version 1.18.0) using its GO and IC tables and the best-match average approach to compare genes. Pesquita *et al.* showed that the best-match average approach performs best [6]. We computed Wang’s similarity, IC-based particularity and SV-based particularity using an in-house implementation of each measure and the August 2013 version of GO. We computed simUI and simGIC similarities using the web tool CMPSim provided by the XLDB research group [41]. CMPSim implements both measures for ChEBI.

### Similarity threshold determination using two groups of similar genes

We first applied our method to determine the similarity threshold for the Biological Processes (BP) using two groups of similar genes. We determined thresholds using first Wang’s and then Lin’s similarity measures.

#### Group determination

We composed two groups of similar genes from two families of the Protein ANalysis THrough Evolutionary Relationships database (PANTHER). The union of the pairs of genes within each family constituted the S distribution. The PANTHER database classifies proteins (and their genes) to facilitate high-throughput analysis [42]. PANTHER families are composed of genes sharing evolutionary history, molecular functions and biological processes annotations, and involvment in the same biological pathways. We assumed that genes belonging to a same PANTHER family share enough features to be considered as involved in similar biological processes. Conversely, we assumed that two genes belonging to two different PANTHER families should not be considered as involved in similar biological processes.

#### Intra-group and inter-group similarity measure

We computed the similarity values for each pair of genes of the first family and for each pair of genes of the second family, and compiled them together in the S distribution. We then computed the N distribution composed of the similarity values between each gene from the first family and each gene from the second family.

#### Similar and non-similar distribution comparison

When comparing the distributions of similar genes (S) to non-similar genes (N), if the minimum value of S is smaller than the maximum value of N, then the S and N distributions overlap and any threshold would lead to FPs or FNs.

**Fig 2:**
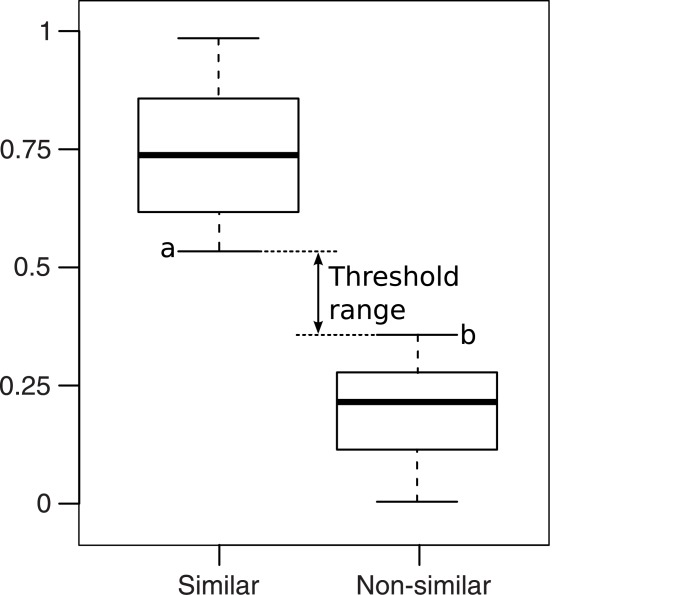
Ideal case of threshold determination. The threshold should be located between the lowest whisker of the similar distribution (a) and the upmost whisker of the non-similar distribution (b).

Fig 2 illustrates the case without overlap, where min(S) = *a*, max(N) = *b* and *a* > *b*. A similarity value greater than *a* means that the genes compared are similar. A similarity value lower than *b* means that the genes compared are non-similar. A similarity value between *a* and *b* means that the genes compared are nearly similar and thus require expert opinion to interpret the result.

**Fig 3:**
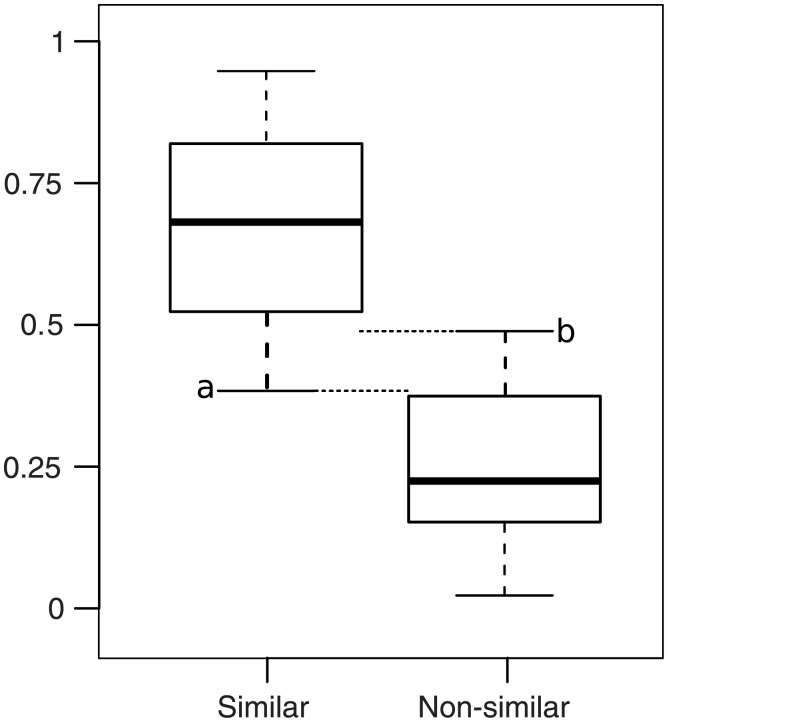
Overlap case of threshold determination. The similar and non-similar boxes overlap. In this case, there are false-positive and false-negative results between the lowest whisker of the similar distribution (a) and the upmost whisker of the non-similar distribution (b).

Fig 3 illustrates the case where the S and N distributions overlap, meaning that there are some FPs (i.e. pairs of genes from N that are non-similar but that have a similarity value greater than a) and FNs (i.e. pairs of genes from S that are similar but have a similarity value lower than b). In this case, a similarity value lower than *a* means that the genes compared are non-similar. A similarity value greater than *b* means that the genes compared are similar. Again, expert opinion would be required to interpret the result in this interval. However, in this case, it is possible to determine the threshold value that minimizes both FP and FN.

We established a general framework that proves suitable to the two cases described in this section. Under this framework, we define three thresholds values:

- *τ*_*S*_ = max(*a*, *b*) is the threshold value above which the two compared genes are similar. There can not be any FP above *τ*_*S*_, but there may be some FN below *τ*_*S*_ if *a* < *b*.
- *τ*_*N*_ = min(*a*, *b*) is the threshold value under which the two compared genes are non-similar. There cannot be any FN below *τ*_*N*_, but there may be some FP above *τ*_*N*_ if *a* < *b*.
- *τ*_*sim*_ is the threshold value located between *τ*_*S*_ and *τ*_*N*_ that that minimizes the proportion of FP and FN. As *τ*_*sim*_ gets closer to *τ*_*S*_, there will be more FN and fewer FP. Conversely, as *τ*_*sim*_ gets closer to *τ*_*N*_, there will be more FP and fewer FN. *τ*_*sim*_ has to be computed using the proportions of FP and FN as the S and N distributions have different sizes.

### Threshold stability study

#### Extension to multiple families

The more groups we build to constitute the S and N distributions, the more reliable the thresholds obtained become. We generalized the above-described process using five groups of similar genes for CC and six groups for BP and MF in order to determine *τ*_*S*_, *τ*_*N*_ and *τ*_*sim*_ for Wang’s and Lin’s measures.

For BP, we computed the S distribution gathering the similarity values of each pair of genes inside six different PANTHER families. We computed the fifteen distributions corresponding to all the combinations of genes similarity values from two of the previous six families. Each of these distributions is composed of the similarity values between each gene from the first family and each gene from the second family. We combined all these inter-family similarity values into a global N distribution.

For MF, we used the same six genes families to compute our S and N distributions, as the PANTHER families are also homogeneous in term of molecular functions.

For CC, we used the genes from five different pathways, each located in a different cellular compartment, to compute our S and N distributions. The lists of genes were borrowed from the Reactome database [43].

#### Robustness of threshold determination

We validated our study using a leave-one-out approach that consisted in successively recomputing the thresholds using all the sets but one. This approach provides an evaluation of threshold stability.

### Generalization

We generalized the approach by applying the method to another semantic measure and another ontology.

#### Particularity threshold

In addition to the similarity thresholds determination, we used the same approach to compute semantic particularity thresholds on BP, CC and MF in order to determine the comparison profile of two genes G1 and G2. The procedure consisted in comparing each value of the triple (Similarity(G1, G2); Particularity(G1, G2); Particularity(G2, G1)) with its respective threshold (noted “+” if the value is greater than the threshold, and “-” otherwise). The results of comparing two genes on their similarity and particularity values can be classified into eight distinct patterns described in Table 1. A comparison should not result in a “+ + +” nor a “- - -” pattern. Indeed, a “+ + +” pattern would mean that the two genes compared share enough features to be considered similar yet, at the same time, that each have enough particular features to both be considered particular. Conversely, a “- - -” pattern would mean that the two genes compared are neither similar nor particular.

**Table 1:** Patterns of similarity and particularity.

Notation	sim(A, B)	par(A, B)	par(B, A)
+ + +	⩾ *τ*_*sim*_	⩾ *τ*_*par*_	⩾ *τ*_*par*_
+ + -	⩾ *τ*_*sim*_	⩾ *τ*_*par*_	< *τ*_*par*_
+ - +	⩾ *τ*_*sim*_	< *τ*_*par*_	⩾ *τ*_*par*_
+ - -	⩾ *τ*_*sim*_	< *τ*_*par*_	< *τ*_*par*_
- + +	< *τ*_*sim*_	⩾ *τ*_*par*_	⩾ *τ*_*par*_
- + -	< *τ*_*sim*_	⩾ *τ*_*par*_	< *τ*_*par*_
- - +	< *τ*_*sim*_	< *τ*_*par*_	⩾ *τ*_*par*_
- - -	< *τ*_*sim*_	< *τ*_*par*_	< *τ*_*par*_

The results of a semantic comparison of gene annotations can be classed into eight macro-patterns according to similarity and particularity values. The first sign is a “+” if the similarity is greater than or equal to the similarity threshold *τ*_*sim*_, or a “-” otherwise. The two other signs depends on the two particularity values, a “+” for a particularity greater than the particularity threshold *τ*_*par*_ or a “-” otherwise.

We applied the threshold determination process described in Fig 1 to obtain a particularity threshold. For the first step, we composed the same gene groups as those used to compute the similarity threshold. For the second step, we computed all the intra-group and inter-group particularity values between all possible pairs of genes. At the third step, we did not consider any FPs nor FNs as genes belonging to the same group can have some degree of particularity even if they are similar. However, knowing the similarity threshold, we computed the proportion of “+ + +” and “- - -” patterns found in the results while particularity threshold varied. For this step, three similarity thresholds were available: *τ*_*N*_, *τ*_*S*_ and *τ*_*sim*_. Let *sim* be the result of a semantic similarity measure between two genes G1 and G2.

- If *sim* is lower than *τ*_*N*_, we can conclude that G1 and G2 are strictly non-similar. Conversely, if *sim* is greater than *τ*_*N*_, we can only conclude that G1 and G2 are possibly similar but with no certainty.
- If *sim* is greater than *τ*_*S*_, we can conclude that G1 and G2 are strictly similar. Conversely, if *sim* is lower than *τ*_*S*_, we only can conclude that G1 and G2 are possibly non-similar but with no certainty.
- Using *τ*_*sim*_ cannot lead to a conclusion with absolute certainty, but it does lead to the smallest number of errors.

Using *τ*_*N*_ can result in a lot of FPs and using *τ*_*S*_ can result in a lot of FNs. Consequently, we computed the particularity threshold *τ*_*par*_ using the similarity threshold *τ*_*sim*_. For step 3c, we summed the “+ + +” and “- - -” proportions for each possible particularity threshold value. The particularity threshold *τ*_*par*_ was the one that minimized this sum.

#### ChEBI

As the threshold determination process is neither specific to GO nor to the previously used measures, we applied our method to another ontology using two other similarity measures. We compared families of molecules using the ChEBI ontology and the simUI and the simGIC similarity measures. We composed our S and N distributions from the pairwise similarities obtained comparing all the children of two ChEBI entities. These entities were two distinct general (i.e. with no common descendants) ChEBI terms, each of which is the parent of numerous specific terms in the ChEBI ontology. This process allowed us to compare two distinct families of molecules.

### Evaluation

The evaluation study involved first quantifying the extent of the changes resulting from using the threshold computed by our method instead of the default 0.5 and then determining whether these changes are biologically relevant.

The first part of this study focused on the changes in the results of the whole HomoloGene database intra-group gene comparisons. HomoloGene is a system that automatically detects homologs, including paralogs and orthologs, among the genes of 21 fully-sequenced eukaryotic genomes [44].

In the second part of this study, we computed the similarity and particularity measures on the well annotated peroxisome proliferator activated receptor (PPAR) multigene family. PPAR*α*, PPAR*β* and PPAR*γ* are involved in different processes [45] as transcription factors. Each member of this family uses the same molecular mechanisms in different metabolic pathways. The family is evolutionarily well conserved [46]. We expected a similarity value above the threshold for BP when comparing PPAR orthologs in several species. However, the ortholog conjecture assumes that orthologs generally share more functions than paralogs. We consequently expected some similarity values below the threshold when comparing PPAR paralogs within a species and between species. The goal was to determine whether our similarity and particularity thresholds lead to biologically more relevant interpretations than the default approach.

## Results and Discussion

### BP similarity threshold using two groups of similar genes

We studied the similarity values obtained when comparing genes known to be functionally close and genes without functional proximity. This study was performed using a hybrid semantic similarity measure (Wang) and a node-based measure (Lin).

**Fig 4:**
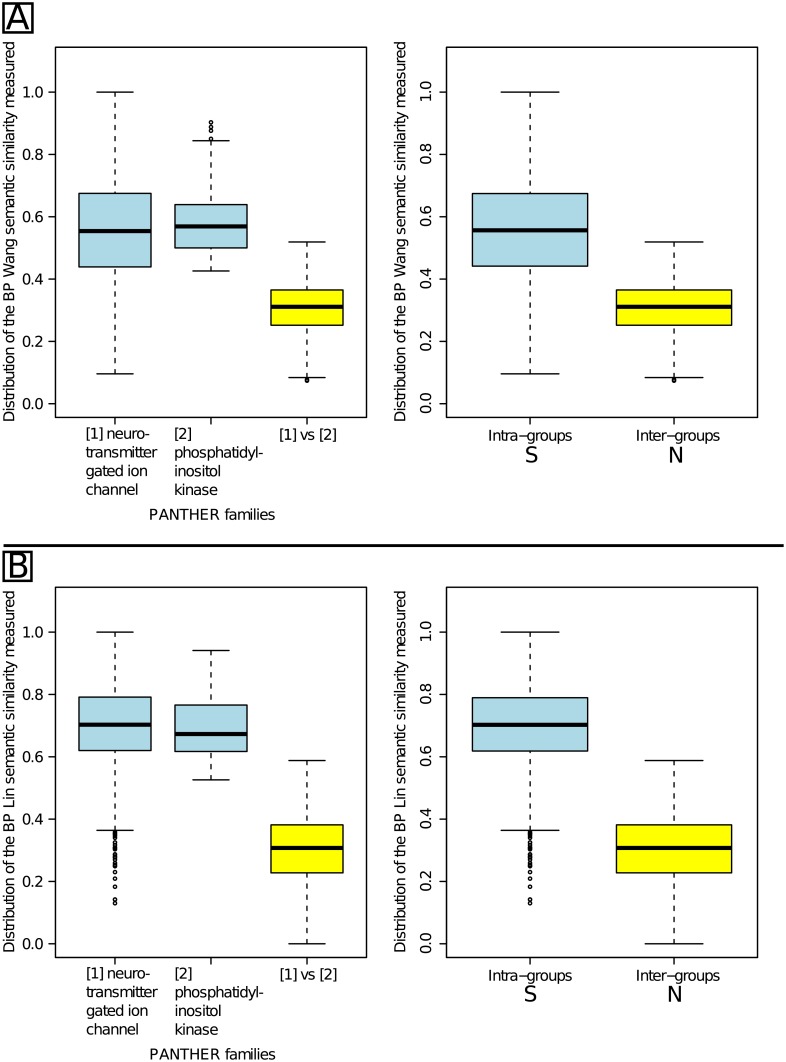
Intra- and inter-family semantic similarity distributions using two families of similar genes. Part A presents the results obtained using Wang’s measure and part B presents the results obtained using Lin’s measure. In both parts, the left side separately presents the two intra-family distributions in blue and the inter-family distribution in yellow. The right side presents the S distribution that gathers all the intra-family similarity values in blue and the N distribution that gathers all the inter-family similarity values in yellow.

Fig 4 presents the distribution of the BP similarity values obtained for two intra-family comparisons and the corresponding inter-family comparisons. The two PANTHER families were “neurotransmitter gated ion channel” (pthr18945) and “tyrosine-protein kinase receptor” (pthr24416).

As expected, similarity values obtained using either Wang’s (Fig 4A) or Lin’s measure (Fig 4B) were significantly higher in the intra-family comparisons than the inter-family comparisons (Welch’s t-tests; see S1 File). We observed an overlap between the S and N distributions, which corresponds to the situation shown in Fig 3. *τ*_*N*_ was located at the lowest whisker of the intra-family S blue box, *i.e.* 0.096 with Wang’s measure and 0.364 with Lin’s measure. *τ*_*S*_ was located at the upmost whisker of the inter-family N yellow box, *i.e.* 0.519 with Wang’s measure and 0.588 with Lin’s measure.

**Fig 5:**
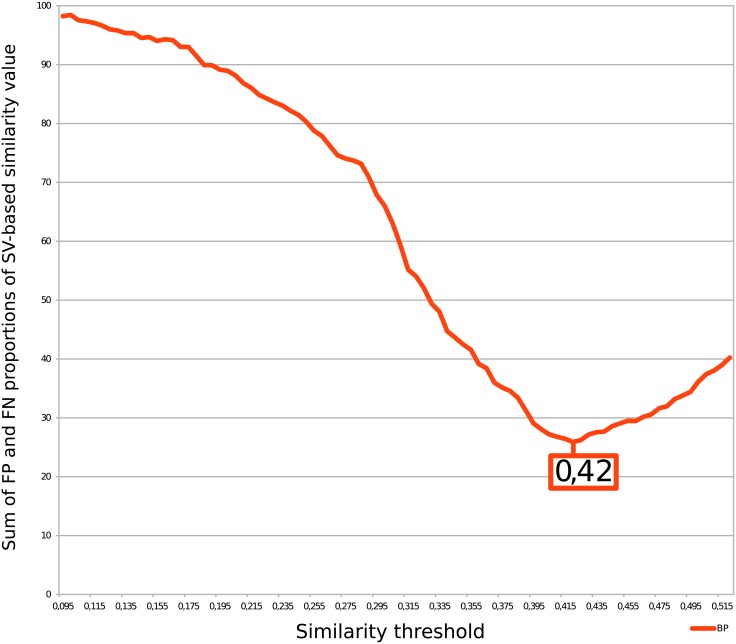
Determination of Wang’s similarity threshold using two families of similar genes. The minimum of false-positive and false-negative proportions gives the similarity threshold (*τ*_*sim*_).

**Fig 6:**
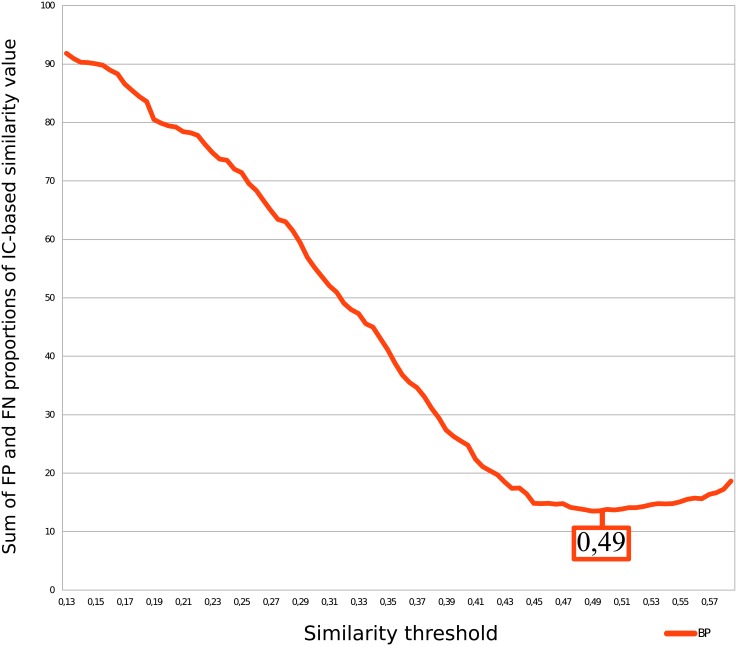
Determination of Lin’s similarity threshold using two families of similar genes. The minimum of false-positive and false-negative proportions gives the similarity threshold (*τ*_*sim*_).

We also determined the optimal similarity threshold value *τ*_*sim*_ that minimizes the sum of FP and FN proportions. Fig 5 reports the results for Wang’s measure and Fig 6 reports the results for Lin’s measure. The minimum ordinate value of the curve of Figs 5 and 6 gives the threshold for BP using Wang’s (0.42) and the Lin’s (0.49) measures, respectively.

### Threshold stability

A threshold determined using only two groups of genes is exposed to bias. In order to obtain a more reliable threshold, we extended the threshold determination process by including the genes from six PANTHER families for BP and MF and the genes from five metabolisms for CC. We then performed a leave-one-out study to assess the stability of the threshold.

#### Extension to multiple families

**Fig 7:**
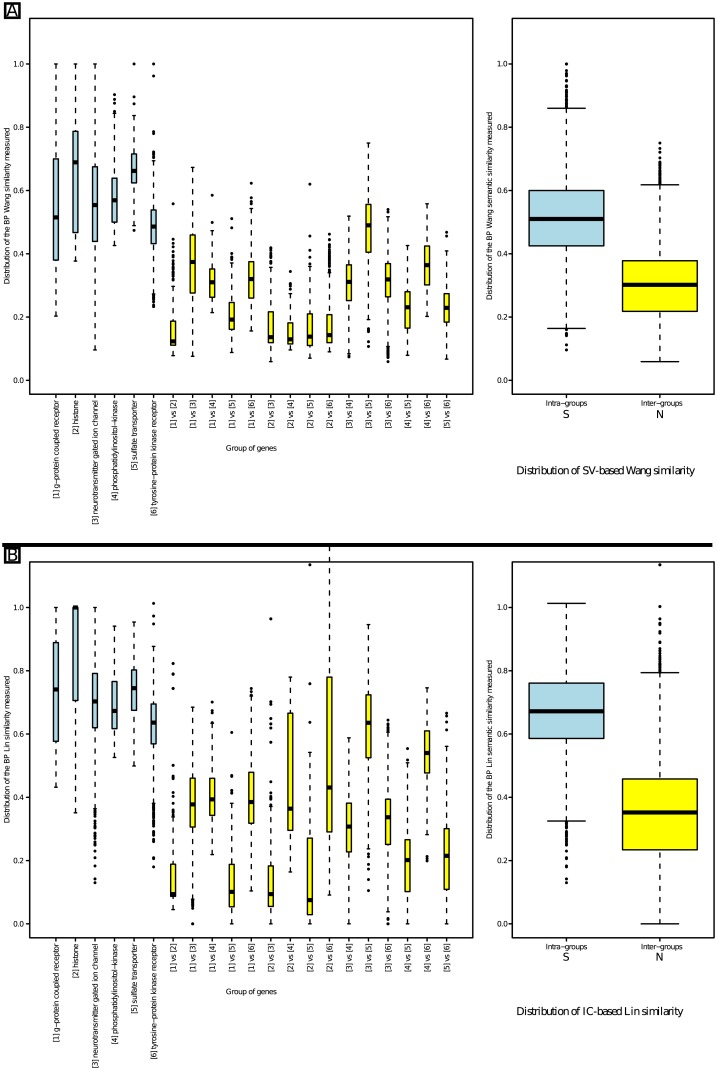
BP distribution of similarity values comparing similar and non-similar genes. Part A gives results using Wang’s similarity measure. Part B gives results using Lin’s similarity measure.

Fig 7 presents the distribution of the BP similarity values obtained for six intra family comparisons and the corresponding fifteen inter-family comparisons. These families were “histone h1/h5 (pthr11467)”, “g-protein coupled receptor” (pthr12011), “neurotransmitter gated ion channel” (pthr18945), “tyrosine-protein kinase receptor” (pthr24416), “phosphatidylinositol kinase” (pthr10048) and “sulfate transporter” (pthr11814). As expected, the similarity values obtained were significantly higher using either Wang’s (Part A) or Lin’s (Part B) measure in the intra-family comparisons than in the inter-family comparisons (Welch’s t-tests; see S2 File). As the S and N distributions overlap, *τ*_*N*_ was located at the lowest whisker of the intra-family S blue box, *i.e.* 0.164 with Wang’s measure and 0.325 with Lin’s measure. *τ*_*S*_ was located at the upmost whisker of the inter-family N yellow box, *i.e.* 0.618 with Wang’s measure and 0.794 with Lin’s measure. These results obtained using six PANTHER families were close to those obtained using two families.

**Fig 8:**
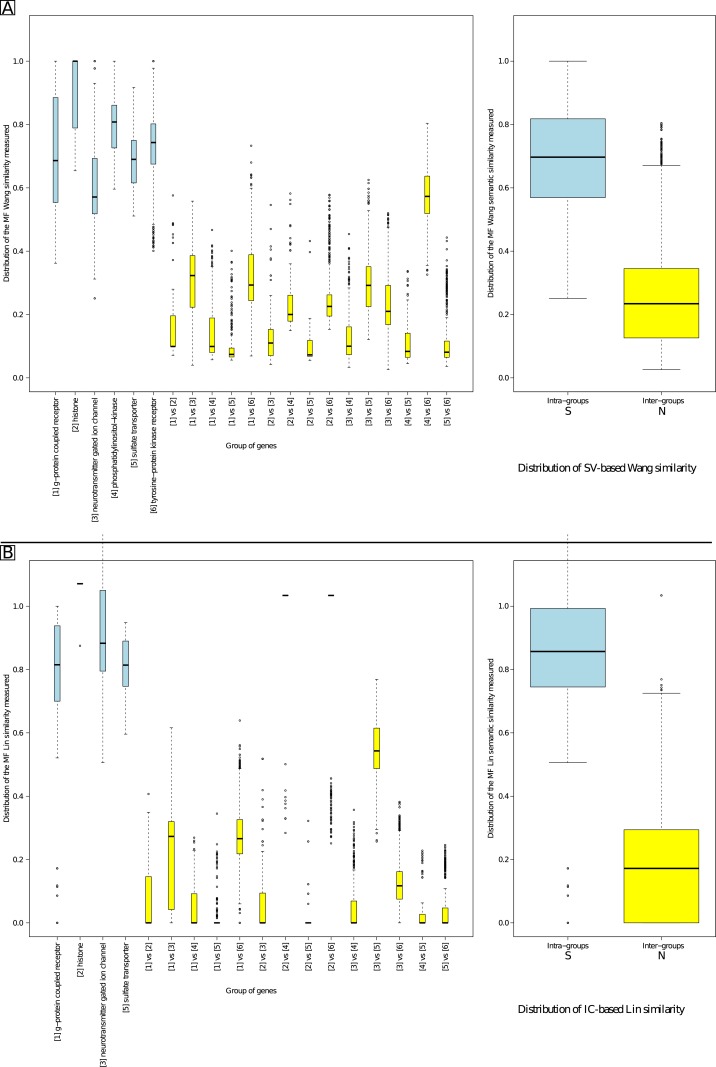
MF distribution of similarity values comparing similar and non-similar genes. Part A gives results using Wang’s similarity measure. Part B gives results using Lin’s similarity measure.

Fig 8 presents the distribution of the MF similarity values obtained for the same six intra-PANTHER family comparisons and the corresponding fifteen inter-family comparisons. Again and as expected, similarity values obtained were significantly higher using Wang’s (Part A) or Lin’s (Part B) measure in the intra-group similarity than the inter-group comparison (Welch’s t-tests; see S3 File). As the S and N distributions overlap, *τ*_*N*_ was located at the lowest whisker of the intra-family S blue box, *i.e.* 0.251 with Wang’s measure and 0.506 with Lin’s measure. *τ*_*S*_ was located at the upmost whisker of the inter-family N yellow box, *i.e.* 0.671 with Wang’s measure and 0.725 with Lin’s measure.

**Fig 9:**
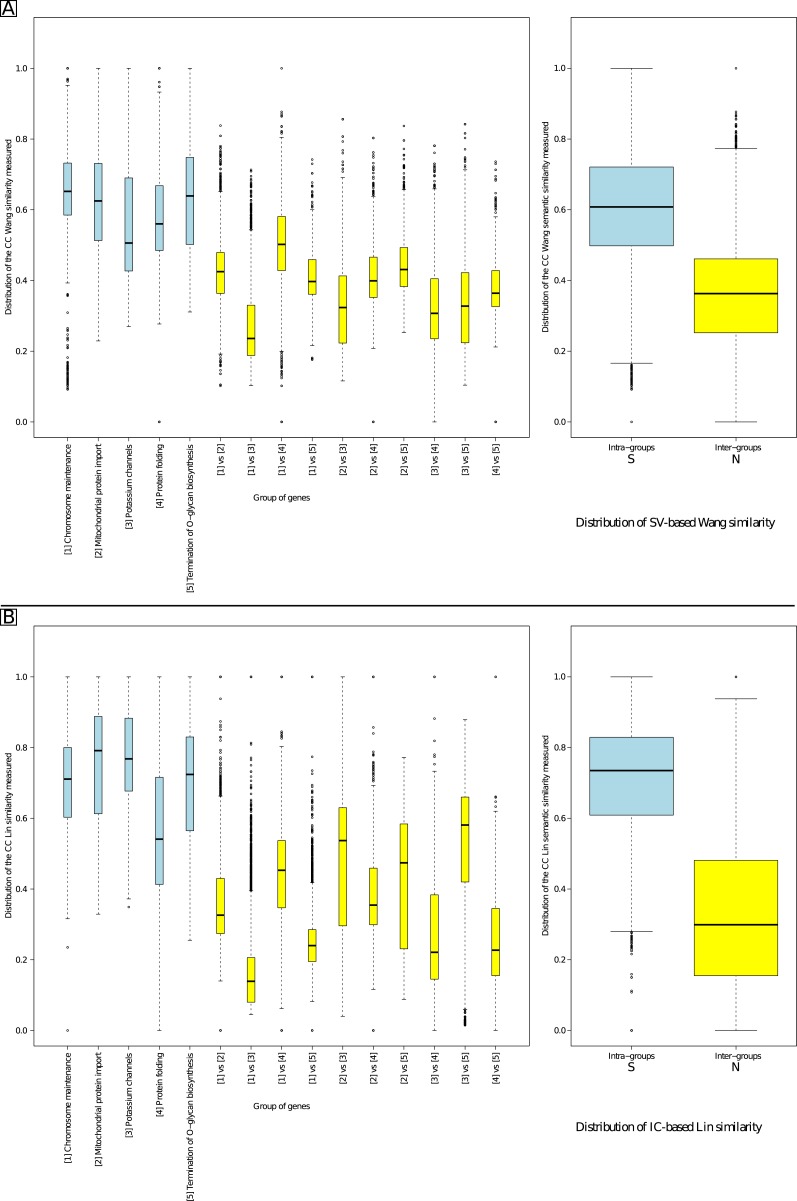
CC distribution of similarity values comparing similar and non-similar genes. Part A gives results using Wang’s similarity measure. Part B gives results using Lin’s similarity measure.

Fig 9 presents the distribution of the CC similarity values obtained for five intra-pathway comparisons and the corresponding ten inter-pathway comparisons. The five pathways chosen were: “chromosome maintenance” (nucleoplasm and nuclear membrane), “mitochondrial protein import” (mitochondrial inter-membrane space, membrane and matrix), “potassium channel” (cellular membrane), “protein folding” (cytosol) and “termination of O-glycan biosynthesis” (Golgi lumen). Similarity values obtained were again significantly higher using either Wang’s (Part A) or Lin’s (Part B) measure in the intra-groups similarity than the inter-group comparison (Welch’s t-tests; see S4 File). As the S and N distributions overlap, *τ*_*N*_ was located at the lowest whisker of the intra-family S blue box, *i.e.* 0.166 with Wang’s measure and 0.28 with Lin’s measure. *τ*_*S*_ was located at the upmost whisker of the inter-family N yellow box, *i.e.* 0.773 with Wang’s measure and 0.938 with Lin’s measure.

**Fig 10:**
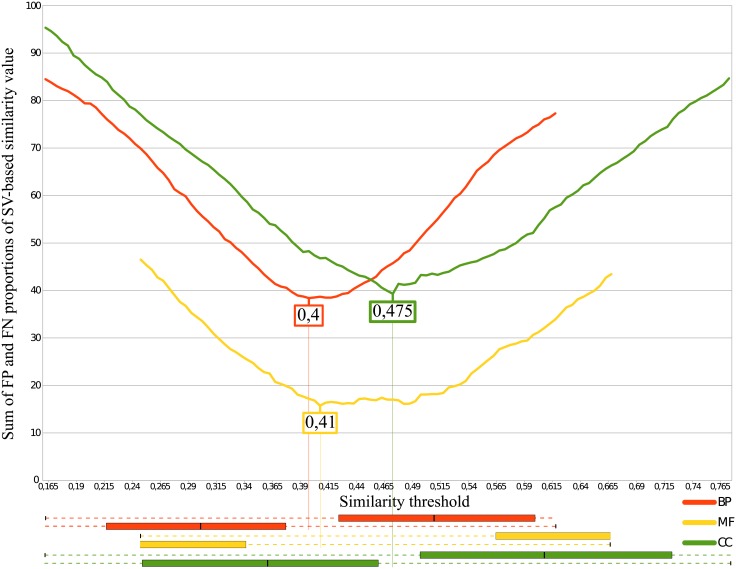
Determination of Wang’s similarity threshold. The minimum of false-positive and false-negative proportions gives the similarity threshold (*τ*_*sim*_). The overlapping parts of the boxplots (between *τ*_*N*_ and *τ*_*S*_) from part A of Figs 7, 8 and 9 are shown in the lower part of the figure. The thresholds are located between the similar and non-similar boxes.

**Fig 11:**
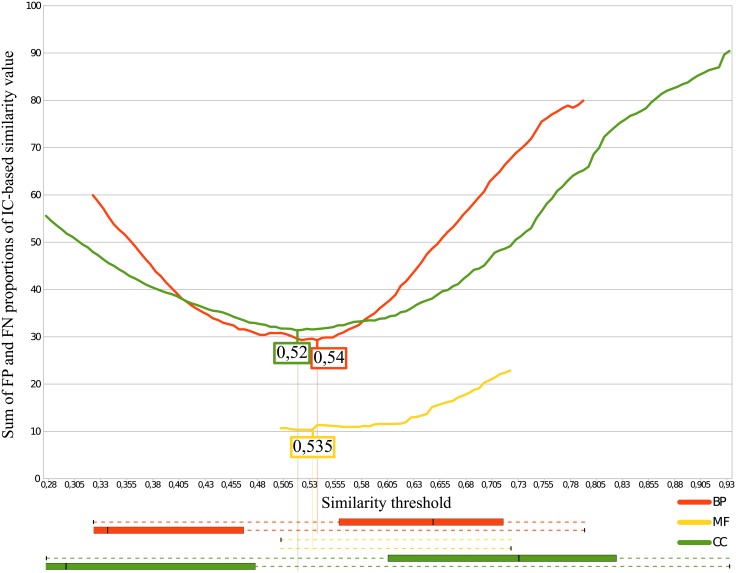
Determination of Lin’s similarity threshold. The minimum of false positive and false negative proportions gives the similarity threshold (*τ*_*sim*_). The overlapping parts of the boxplots (between *τ*_*N*_ and *τ*_*S*_) from part B of Figs 7, 8 and 9 are shown in the lower part of the figure. The thresholds are located between the similar and non-similar boxes.

In each previous case, the S and N distributions overlapped so defining a threshold in this interval yields some FPs and some FNs. We determined the optimal similarity threshold value that minimizes the sum of FP and FN proportions. Fig 10 reports the results for Wang’s SV-based measure and Fig 11 reports the results for Lin’s IC-based measure. The minimum ordinate value of each curve of Figs 10 and 11 gives the threshold for BP, MF and CC using Wang’s and Lin’s measures, respectively. Table 2 summarizes the values obtained for the boxplots (Figs 7, 8 and 9 giving *τ*_*S*_ and *τ*_*N*_) and the threshold variation curves (Figs 10 and 11 giving *τ*_*sim*_). These similarity thresholds differed according to similarity measure used. They also differed between BP, MF and CC. This can be explained by the different level of complexity between these three branches [10]. It is possible to use one of the three proposed thresholds (*τ*_*N*_, *τ*_*S*_ and *τ*_*sim*_) depending on the accuracy needed to interpret the semantic similarity results. None of these thresholds is equal to the intuitive “default” threshold of 0.5.

**Table 2:** Semantic similarity thresholds for Wang’s and Lin’s measures.

	Wang			Lin		
	*τ*_*N*_ Genes are not similar under	*τ*_*S*_ Genes are similar above	*τ*_*sim*_ Threshold minimizing FP and FN	*τ*_*N*_ Genes are not similar under	*τ*_*S*_ Genes are similar above	*τ*_*sim*_ Threshold minimizing FP and FN
BP	0.164	0.618	0.4	0.325	0.794	0.54
MF	0.251	0.671	0.41	0.506	0.725	0.535
CC	0.166	0.773	0.475	0.28	0.938	0.52

For each measure, *τ*_*N*_ and *τ*_*S*_ respectively give the value of the lowest whisker of the blue box and the upmost whisker of the yellow box of the boxplots reported in the Figs 7, 8 and 9. For each measure, *τ*_*sim*_ is the threshold value that minimizes the proportions of false-positive and false-negative results, corresponding to the minimum ordinate of the curves in Figs 10 and 11.

S5 File provides a detailed How To guide to compute a similarity threshold, taking as example the computation of BP similarity threshold using Wang’s measure.

#### Robustness of threshold determination

In order to study the robustness of our optimization, we successively removed one gene set from our datasets and re-computed the similarity threshold. We performed this analysis on BP, MF and CC. Tables 3 and 4 present the results for Wang’s and Lin’s measures, respectively, giving the *τ*_*sim*_ and the FP and FN proportions for each complete dataset and for all the groups of a dataset except one. The thresholds varied slightly over the different datasets.

**Table 3:** Similarity threshold variations considering full and partial datasets (Wang’s measure).

Set	*τ*_*sim*_	FN(%)	FP(%)
BP set	0.4	18.688	19.7
BP set without histone	0.42	23.429	16.372
BP set without g-protein coupled receptor	0.405	16.103	17.626
BP set without neurotransmitter gated ion channel	0.4	19.276	17.03
BP set without tyrosine-protein kinase receptor	0.435	27.708	14.451
BP set without phosphatidylinositol-kinase	0.4	18.954	19.908
BP set without sulfate transporter	0.42	23.642	14.784
MF set	0.41	1.602	14.15
MF set without histone	0.41	1.625	14.763
MF set without g-protein coupled receptor	0.41	1.831	13.842
MF set without neurotransmitter gated ion channel	0.49	4.599	8.668
MF set without tyrosine-protein kinase receptor	0.41	2.666	12.419
MF set without phosphatidylinositol-kinase	0.41	1.625	12.666
MF set without sulfate transporter	0.41	1.63	14.993
CC set	0.475	17.864	21.443
CC set without chromosome maintenance	0.475	27.342	20.251
CC set without mitochondrial protein import	0.475	18.041	21.114
CC set without potassium channels	0.515	15.987	17.133
CC set without protein folding	0.475	17.417	19.082
CC set without termination of O-glycan biosynthesis	0.475	17.867	21.717

This table summarizes the similarity thresholds *τ*_*sim*_ obtained considering each complete dataset or all the groups of a dataset except one, when using Wang’s similarity measure. The numbers given for FP and FN are the proportions of false-positives and false-negatives that the threshold admits in the comparison results.

**Table 4:** Similarity threshold variations considering full and partial datasets (Lin’s measure).

Set	*τ*_*sim*_	FN(%)	FP(%)
BP set	0.54	16.401	12.88
BP set without histone	0.54	16.465	12.326
BP set without g-protein coupled receptor	0.525	14.101	16.081
BP set without neurotransmitter gated ion channel	0.525	15.556	15.887
BP set wihout tyrosine-protein kinase receptor	0.54	14.403	12.969
BP set without phosphatidylinositol-kinase	0.525	14.687	14.071
BP set without sulfate transporter	0.54	16.633	12.144
MF set	0.535	2.514	7.799
MF set without histone	0.535	2.584	5.756
MF set without g-protein coupled receptor	0.565	0.9	9.016
MF set without neurotransmitter gated ion channel	0.535	4.258	8.661
MF set without tyrosine-protein kinase receptor	0.535	2.514	7.849
MF set without phosphatidylinositol-kinase	0.535	2.514	7.817
MF set without sulfate transporter	0.52	2.431	7.265
CC set	0.52	11.838	19.538
CC set without chromosome maintenance	0.545	15.222	19.971
CC set without mitochondrial protein import	0.52	12.266	17.596
CC set without potassium channels	0.52	16.347	18.905
CC set without protein folding	0.52	8.072	20.313
CC set without termination of O-glycan biosynthesis	0.52	11.641	18.463

This table summarizes the similarity thresholds obtained considering each complete dataset or all the groups of a dataset except one, when using Lin’s similarity measure. The numbers given for FP and FN are the proportions of false-positives and false-negatives that the threshold admits in the comparison results.

BP similarity threshold varied between 0.4 and 0.435. MF similarity threshold remained stable at 0.41, except when not taking into account the family of genes related to neurotransmitter gated ion channels (0.49). CC similarity threshold was between 0.475 and 0.515.

The MF case diverged from BP and CC on its similarity (FP + FN proportions) curve. Indeed, the minimum value of 0.41 was located at the extreme left of a part of the curve where (FP + FN proportions) varied slightly. Consequently, leaving out the “neurotransmitter gated ion channels” dataset that was causing this specific minimum position greatly affected the threshold. However, some perspective is needed: first, there was a relatively long interval in which the sum of FP and FN remained low, and second, the minimum of 0.49 obtained without the “neurotransmitter gated ion channels” set was located at the opposite part of this range of stability.

Considering Figs 10 and 11, the minimum ordinate value of the sums FP + FN proportions was in each case located in a relatively large range within which the ordinate varied only slightly. Consequently, we concluded that the similarity could be located in the range where the sum of the FP and FN proportions varied the least. Finally, note that each threshold presented here was source of errors (FP and FN) in the proportions described in Tables 3 and 4.

#### Generalization

We applied our threshold determination method to obtain a particularity threshold on GO and a similarity threshold for two measures on the ChEBI ontology.

#### Particularity threshold

We used the semantic particularity measure of Bettembourg *et al.* with SV and IC, respectively, to compute the particularity values for the same genes used in the similarity study. The variation of the “+ + +” and “- - -” profiles in our datasets was studied using the similarity threshold *τ*_*sim*_ obtained in the previous section and sampling the value of *τ*_*par*_, the particularity threshold. Table 5 gives the particularity thresholds (*τ*_*par*_) minimizing the sum of “+ + +” and “- - -” patterns for SV-based and IC-based approaches. S6 File presents the values that supported the thresholds determination.

**Table 5:** Semantic SV-based and IC-based particularity thresholds.

	SV-based particularity threshold	IC-based particularity threshold
BP	0.515	0.68
MF	0.485	0.66
CC	0.335	0.6

These thresholds minimize the proportions of non-informative “+ + +” or “- - -” patterns according to Table 1.

These thresholds differed between BP, MF and CC and between approaches. We performed the leave-one-out study in order to assess stability of the particularity threshold by removing one gene set from our datasets and re-computing the particularity threshold. This analysis was performed on BP, MF and CC. We obtained *τ*_*par*_ and the proportions of non-informative “+ + +” and “- - -” cases for each complete dataset and for all the groups of a dataset except one. The thresholds varied slightly among the different datasets. BP particularity threshold was between 0.49 and 0.515. MF particularity threshold was between 0.35 and 0.485. CC particularity threshold was between 0.28 and 0.335. S6 File provides the detailed results of the leave-one-out study using SV and IC as informativeness measures.

With both SV-based and IC-based approaches, the minimum ordinate value of the sums “+ + +” + “- - -” was located in a relatively large range within which the ordinate varied only slightly. Consequently, we concluded that the particularity thresholds should be located in the range where the sum of the “+ + +” and “- - -” proportions varied the least.

#### simUI and simGIC thresholds for ChEBI molecular entities

**Fig 12:**
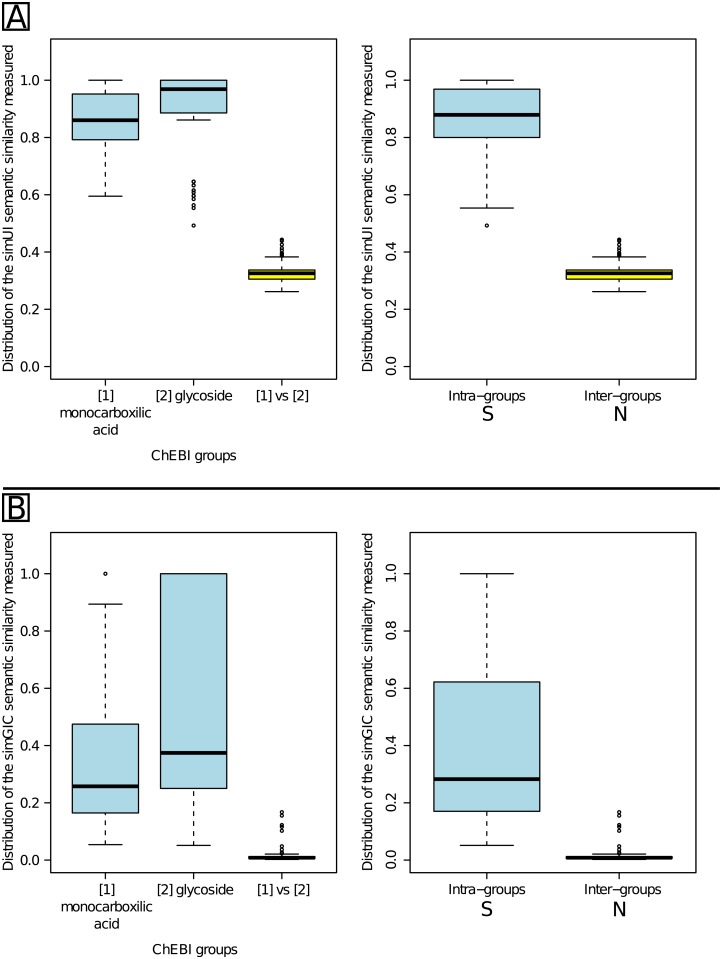
Distribution of similarity values comparing similar and non-similar ChEBI entities. Part A gives results using the simUI similarity measure. Part B gives results using the simGIC similarity measure. The S and N distributions did not overlap. For both measures, *τ*_*sim*_ was between *τ*_*S*_ (lowest whisker of the intra-family S blue box) and *τ*_*N*_ (upmost whisker of the inter-family N yellow box).

Fig 12 presents the distribution of the similarity values obtained for the intra and inter-groups comparisons using the two ChEBI groups composed of children of “monocarboxylic acid” (chebi:25384) and “glycoside” (chebi:24400). As expected, similarity values obtained were significantly higher using either the simUI (Part A) or simGIC (Part B) measures in the intra-group comparisons than the inter-group comparisons (Welch’s t-tests; see S7 File). Unlike the results obtained on the GO, the S and N distributions did not overlap. We were this time in the situation described by Fig 2. Consequently, *τ*_*S*_ was located at the lowest whisker of the intra-family S blue box, *i.e.* 0.554 for simUI and 0.051 for simGIC. *τ*_*N*_ was located at the upmost whisker of the inter-family N yellow box, *i.e.* 0.383 for simUI and 0.021 for simGIC. It is possible to choose any value between *τ*_*N*_ and *τ*_*S*_ as similarity threshold. Note that weighting by the IC in the simGIC measure resulted in a very low threshold.

### Evaluation

We evaluated the GO similarity and particularity thresholds in two different use-cases. First, we compared the interpretation of the results of semantic measures performed on homolog genes using a default threshold of 0.5 vs our new thresholds. Second, we studied whether the thresholds determined via our new method led to biologically-relevant interpretations.

#### Large-scale evaluation of the impact of threshold changes

We evaluated the impact of our new GO similarity and particularity thresholds over a large dataset characterization. We compared the distribution of semantic measures results among the different patterns proposed in Table 1 for the whole HomoloGene database considering an arbitrary 0.5 threshold and our new method thresholds. Tables 6, 7 and 8 summarize the results for BP, MF and CC, respectively. They provide the number of pairs of genes changing from one pattern of Table 1 to another using *τ*_*sim*_ and *τ*_*par*_ instead of the default value of 0.5. We have not distinguished the “+ + -” and “+ - +” categories nor the “- + -” and “- - +” categories as the order of particularity values in the results of this study is meaningless. All categories of the pattern described in Table 1 were impacted by the change of threshold. As the new thresholds were different between BP, MF and CC, the transitions observed were also different. For example, the number of “+ + -” increased for BP but decreased for MF and CC. However, in all cases, the greatest size increase concerned the “+ + - or + - +” category, at +26.2%, +18.5% and + 36.7% for BP, MF and CC, respectively. The number of “+ + +” and “- - -” cases, that are the least-informative cases, decreased for BP (-11.2%) and MF (-34.8%) but increased for CC (+49%). This situation can be explained by the fact that the CC particularity threshold of 0.335 was the lowest of all the computed thresholds, making the increase of “+ + +” cases more important than the decrease of the “- - -” cases. Furthermore, the average number of CC terms that annotate a gene in HomoloGene was only 1.38 against 2.45 for BP and 1.63 for MF. Consequently, the similarity and particularity values measured on HomoloGene were less reliable for CC than for BP and MF. This situation could be attributed to a lack of CC annotations in our dataset. However, in the three branches of GO, the proportions of the least-informative cases were low at just 1.62%, 0.39% and 1.30% for BP, MF and CC, respectively. Overall, the change of thresholds deeply impacted the distribution the HomoloGene intra-group comparison results between the different patterns.

**Table 6:** Evolution in patterns in results on HomoloGene intra-group BP comparisons.

BP↱	+ - -	+ + - or + - +	+ + +	- + +	- + - or - - +	- - -	Total using 0.5 thresholds
+ - -	268,471	0	0	0	0	0	268,471
+ + - or + - +	1,780	54,168	0	0	0	0	55,948
+ + +	7	270	2,623	0	0	0	2,900
- + +	2	154	2,254	10,374	304	1	13,089
- + - or - - +	177	16,027	0	0	32,578	102	48,884
- - -	2,883	0	0	0	0	1,401	4,284
Total using new thresholds	273,320	70,619	4,877	10,374	32,882	1,504	T = 393,576

Numbers of pairs of genes changing from one pattern to another when considering our optimal similarity and particularity thresholds instead of the default value of 0.5. The most important transition consists in 16,027 results moving from the “- + - or - - +” category (size decreased by 32.7%) to the “+ + - or + - +” category (size increased by 26.2%). The new thresholds give more “+ + +” results but fewer “- - -” results. Globally, the sum of the numbers of the “+ + +” and “- - -” patterns has decreased (-11.2%).

**Table 7:** Evolution in patterns in results on HomoloGene intra-group MF comparisons.

MF↱	+ - -	+ + - or + - +	+ + +	- + +	- + - or - - +	- - -	Total using 0.5 thresholds
+ - -	377,017	2,197	14	0	0	0	379,228
+ + - or + - +	0	37,680	56	0	0	0	37,736
+ + +	0	0	666	0	0	0	666
- + +	0	0	297	8,507	0	0	8,804
- + - or - - +	0	4,738	15	34	12,953	0	17,740
- - -	1,189	87	0	0	25	672	1,973
Total using new thresholds	378,206	44,702	1,048	8,541	12,978	672	T = 446,147

Numbers of pairs of genes changing from one pattern to another when considering our optimal similarity and particularity thresholds instead of the default value of 0.5. After the change of threshold, the most important transition consists in 4,738 results moving from the “- + - or - - +” category (size decreased by 26.8%) to the “+ + - or + - +” category (size increased by 18.5%). The new thresholds give more “+ + +” results but fewer “- - -” results. Globally, the sum of the numbers of the “+ + +” and “- - -” patterns has decreased (-34.8%).

**Table 8:** Evolution in patterns in results on HomoloGene intra-group CC comparisons.

CC↱	+ - -	+ + - or + - +	+ + +	- + +	- + - or - - +	- - -	Total using 0.5 thresholds
+ - -	250,826	25,089	948	0	0	0	276,863
+ + - or + - +	0	67,349	2,103	0	0	0	69,452
+ + +	0	0	1,237	0	0	0	1,237
- + +	0	0	104	2,746	0	0	2,850
- + - or - - +	0	2,292	90	1,191	19,956	0	23,529
- - -	118	196	34	69	470	369	1,256
Total using new thresholds	250,944	94,926	4,516	4,006	20,426	369	T = 375,187

Numbers of pairs of genes changing from one pattern to another when considering our optimal similarity and particularity thresholds instead of the default value of 0.5. After the change of threshold, the most important transition consists in 25,089 results moving from the “+ - -” category (size decreased by 9.4%) to the “+ + - or + - +” category (size increased by 36.7%). The new thresholds give more “+ + +” results but fewer “- - -” results. Globally, the sum of the numbers of the “+ + +” and “- - -” patterns has increased (+49%).

#### Relevance of the method on the PPAR multigene family

We measured similarity and particularity values of PPAR*α*, PPAR*β* and PPAR*γ* between six species. S8 File provides two tables reporting the results of this study for BP and MF, respectively. Each gene was only annotated by one or two CC terms, so we kept CC results out of this study. All our similarity values were greater than *τ*_*sim*_. Consequently, in order to emerge similarity differences between orthologs and paralogs, we had to use *τ*_*S*_. This threshold guarantees that the results above it indicate two similar genes. However, the only conclusion that can be inferred for the gene comparisons resulting in values between *τ*_*sim*_ and *τ*_*S*_ is that there is doubt over whether these genes are similar. The results of inter-orthologs comparisons systematically matched a “+ - -” pattern, as expected. In contrast, the results of inter-paralog comparisons included some values lower than *τ*_*S*_ and greater than *τ*_*par*_, resulting in “+ + -”, “- + -” and “- - +” patterns. In a recent paper, Thomas *et al.* “strongly encourage careful consideration of the interpretations” of GO-related analysis [47]. Consequently, the only possible conclusion here is that the actual state of the PPAR annotation is consistent with the ortholog conjecture, according to a similarity and a particularity measure, using our new thresholds.

### Limitations

As in any annotation-related domain, the threshold determination for a semantic measure is limited by the number of annotations available. There is strong variation in the quantity, granularity and reliability of annotations between different species and different metabolisms, which make it difficult to determine a good threshold when the domain of interest has few annotations. However in such cases, the results of a semantic similarity or particularity measure would not be accurate anyway.

The appropriate choice of “S” and “N” distributions is crucial to the threshold determination process, and it hinges on having some degree of knowledge in the domain of interest. The more these distributions differ from the data to interpret using the threshold, the less accurate this threshold will be.

These two limitations can co-occur if studying a poorly-annotated and little-known species using a threshold obtained from a better-known but not-so-close species.

### Generic method and domain-dependent thresholds

We computed thresholds for several semantic measures. We used them to interpret data from different mammal species. The gene groups used to compute the thresholds were related to six different families (BP and MF thresholds) and five pathways located in a different cell compartment (CC threshold). We believe that these thresholds are more relevant for the comparison of any mammal genes than the arbitrary threshold of 0.5 used to date.

We do not claim that these thresholds are universal. It is preferable to recompute the thresholds in order to compare genes for other species or simply to use thresholds that are up-to-date with the evolution of GO and GOA.

Overall, even if the thresholds are domain-dependent, our threshold computation method can be applied to any domain. It only requires some degree of domain expertise to build the most relevant “S” and “N” distributions. Once a threshold is determined with the help of an expert to compose the relevant datasets, the leave-one-out study indicates that the threshold is applicable to other similar datasets and is in this regard application-independent. However, the user should consider whether the original datasets are still relevant in their own application context (which may be different from the context used to formulate the threshold).

## Conclusion

Here we propose a method for determining a threshold for the interpretation of values obtained with semantic measures. We applied this method to obtain the similarity and particularity thresholds for BP, MF and CC branches of GO and the similarity threshold for the ChEBI ontology. These new thresholds provide new insight on semantic measure results. Using the new thresholds, we showed that the results of comparisons in the HomoloGene database were classified into very different patterns. These new thresholds also better separated orthologs and paralogs in the multigene PPAR family. The new thresholds we proposed are not absolute. As the curves used to define them were rather flat around the minima, we can pick our thresholds from within a relatively large range. The precise threshold values proposed here are only the minimum values of this range. Furthermore, a threshold value should be considered in its biological context and warrants revaluation according to this context and to evolutions in GO and GOA and the semantic measure used.

## Supporting Information

S1 File Welch’s t-test results on the comparison of the Fig 4 BP similarity boxes. (TXT)

S2 File Welch’s t-test results on the comparison of the Fig 7 BP similarity boxes. (TXT)

S3 File Welch’s t-test results on the comparison of the Fig 8 MF similarity boxes. (TXT)

S4 File Welch’s t-test results on the comparison of the Fig 9 CC similarity boxes. (TXT)

S5 File How To guide to compute a BP similarity threshold. (PDF)

S6 File Two figures and two tables presenting the results of the particularity threshold computation. (PDF)

S7 File Welch’s t-test results on the comparison of the Fig 12 ChEBI similarity boxes. (TXT)

S8 File Two tables presenting the results of SV-based BP and MF similarity and particularity measured between orthologs and paralogs of the PPAR family. (PDF)
