# Supplementary material for: Optimal Threshold Determination for Interpreting Semantic Similarity and Particularity: Application to the Comparison of Gene Sets and Metabolic Pathways Using GO and ChEBI
Source: PLoS One. 2015 Jul 31;10(7):e0133579. doi: 10.1371/journal.pone.0133579 (PMC4521860; doi:10.1371/journal.pone.0133579)
Supplement: S5 File — (PDF) [file pone.0133579.s005.pdf]

# How to compute a semantic similarity threshold

Charles Bettembourg, Christian Diot, Olivier Dameron

## Abstract

The analysis of gene annotations related to Gene Ontology plays an important role in the interpretation of high-throughput experiments results. This analysis typically involves semantic similarity and particularity measures that quantify the importance of the Gene Ontology annotations. In our paper entitled "Interpretation of semantic similarity and particularity for functional analysis of gene set", we propose a new method to compute thresholds supporting the interpretation of the results of a semantic comparison. Indeed, there was no sound method supporting the interpretation of the similarity and particularity values in order to determine whether two genes are similar or whether one gene has some significant particular function. Interpretation is frequently based either on an implicit threshold, or an arbitrary one (typically 0.5). Our method determines the optimal similarity threshold by minimizing the proportions of false-positive and false-negative similarity matches using distributions of the similarity values of pairs of similar genes and pairs of non-similar genes. In this "How to", we explain the process step by step for determining one of the thresholds computed in our paper: the Biological Process threshold using Wang's similarity measure.

# Similarity threshold determination process

This process is composed of three steps:

1. Define at least two different groups of similar genes for species of interest.

2. (a) In each group, compute the similarities between each pair of genes (i.e. the intra-group similarities). Gather all the similarity results to obtain an S distribution of similar genes.

   (b) Compute the similarities between each combination of a gene from the first group and a gene from a second group (i.e. the inter-group similarities). Gather all the similarity results to obtain an N distribution of non-similar genes.

3. If the S and N distributions have no overlap between the data ranges (min, max), define the threshold $\tau_{sim}$ using any value between $\tau_S$ (the lowest value of S) and $\tau_N$ (the highest value of N). Else, there are some false negatives (FN) and some false positives (FP):

   (a) Compute the FN proportion of FN in the S distribution for all samples of the similarity threshold between $\tau_N$ to $\tau_S$. In this step, consider every value under the similarity threshold as a FN.

   (b) Compute the FP proportion of FP in the N distribution for all samples of the similarity threshold between $\tau_N$ to $\tau_S$. In this step, consider every value above the similarity threshold as a FP.

   (c) For each possible threshold value, sum the FN and FP proportions obtained in steps 3a and 3b. The similarity threshold $\tau_{sim}$ is the threshold that minimizes this sum.

# Step 1: Define at least two different groups of similar genes for species of interest

Here we need biological knowledge to compose groups of genes that we know similar. As we focus on biological processes, we propose to use PANTHER families. The PANTHER families are composed of genes sharing an evolutionary history, molecular functions and biological processes annotations, and an involvment in the same biological pathways. Consequently, we assumed that genes belonging to a same PANTHER family share enough features to be considered as being involved in similar biological processes. Inversely, we assumed that two genes belonging to two different PANTHER families should not be considered as being involved in similar biological processes.

We need a minimum of two gene groups to compute a threshold but considering more groups increases threshold robustness. In this document, we will use six PANTHER families as groups of similar genes. We select families involved in different biological processes and having at least 10 genes, with no gene common between the selected families. These families are:

- “histone h1/h5 (pthr11467)”
- “g-protein coupled receptor” (pthr12011)
- “neurotransmitter gated ion channel” (pthr18945)
- “tyrosine-protein kinase receptor” (pthr24416)
- “phosphatidylinositol kinase” (pthr10048)
- “sulfate transporter” (pthr11814)

Table 1 gives the group of genes for each family.

Table 1: **Genes of six PANTHER families**

| PANTHER family | Genes |
| --- | --- |
| histone h1/h5 (pthr11467) | H1FOO HIST1H1A HIST1H1B HIST1H1C HIST1H1D HIST1H1E HIST1H1T H1FX H1F0 H1FNT |
| g-protein coupled receptor (pthr12011) | GLP1R CALCRL GPR64 GPR111 GPR112 GPR113 GPR114 GPR115 GPR116 GPR125 GPR56 CRHR1 EVA1C ELTD1 EMR2 CELSR3 BAI3 BAI2 PTH2R CD97 GIPR PTH1R GPR110 LPHN2 GPR128 EMR1 GPR133 GHRHR SCTR GCGR GPR97 LPHN1 VIPR1 GPR144 GPR124 GLP2R VIPR2 ADCYAP1R1 LPHN3 EMR3 CRHR2 GPR123 GPR126 |
| neurotransmitter gated ion channel (pthr18945) | CHRNE HTR3A CHRND CHRNB4 GABRG1 GLRA4 GABRA6 GABRA3 GABRR2 GABRB3 GABRQ GABRD HTR3D CHRNA5 CHRNA9 CHRNA6 CHRNA2 GABRE GLRA3 CHRNA7 GABRA4 GLRB CHRNA10 CHRNG GABRG2 GABRB1 HTR3B CHRNB1 CHRFAM7A CHRNB2 GABRB2 GABRA2 HTR3C GABRG3 CHRNA4 GABRA5 CHRNB3 CHRNA3 ZACN GABRP GABRR1 GABRA1 CHRNA1 HTR3E GLRA2 GLRA1 |
| tyrosine-protein kinase receptor (pthr24416) | MST1R DDR2 ROS1 NTRK2 LMTK2 NTRK1 ERBB2 MET NTRK3 KIT FGFR3 EPHA4 EPHB1 EPHB4 EPHA5 EPHB3 ALK FGFR4 IGF1R AXL PDGFRA EPHA6 RET EPHA1 CSF1R MERTK TYRO3 FLT3 EGFR EPHA7 ERBB4 ERBB3 FGFR2 KDR FLT4 PDGFRB FLT1 LTK EPHB2 EPHA8 EPHA3 EPHA2 EPHA10 INSR TEK TIE1 INSRR EPHB6 DDR1 MUSK RYK FGFR1 ROR2 ROR1 |
| phosphatidylinositol kinase (pthr10048) | PI4KA PIK3CB PIK3CA PIK3CG PIK3C2G PI4KB PIK3C2A PIK3CD PIK3C2B PIK3C3 |
| sulfate transporter (pthr11814) | SLC26A7 SLC26A5 SLC26A4 SLC26A1 SLC26A2 SLC26A11 SLC26A8 SLC26A9 SLC26A3 SLC26A10 SLC26A6 |

Each PANTHER family of this table is one group of similar genes used to compute a BP threshold.

# Step 2: Semantic similarity computation

## Intra-group similarity

At this step, we compute similarities between each pair of genes within each group. For example, for the group "phosphatidylinositol kinase" (pthr10048), we have to compute all pairwise similarities for the couples of genes given in Table 2. The result of these similarity computation gives a first S distribution.

Table 2: **Couple of genes for which we have to compute pairwise similarity for phosphatidylinositol kinase family**

| phosphatidylinositol kinase intra-group couple of genes |
|---|
| (PI4KA, PIK3CB) (PI4KA, PIK3CA) (PI4KA, PIK3CG) (PI4KA, PIK3C2G) (PI4KA, PI4KB) (PI4KA, PIK3C2A) (PI4KA, PIK3CD) (PI4KA, PIK3C2B) (PI4KA, PIK3C3) (PIK3CB, PIK3CA) (PIK3CB, PIK3CG) (PIK3CB, PIK3C2G) (PIK3CB, PI4KB) (PIK3CB, PIK3C2A) (PIK3CB, PIK3CD) (PIK3CB, PIK3C2B) (PIK3CB, PIK3C3) (PIK3CA, PIK3CG) (PIK3CA, PIK3C2G) (PIK3CA, PI4KB) (PIK3CA, PIK3C2A) (PIK3CA, PIK3CD) (PIK3CA, PIK3C2B) (PIK3CA, PIK3C3) (PIK3CG, PIK3C2G) (PIK3CG, PI4KB) (PIK3CG, PIK3C2A) (PIK3CG, PIK3CD) (PIK3CG, PIK3C2B) (PIK3CG, PIK3C3) (PIK3C2G, PI4KB) (PIK3C2G, PIK3C2A) (PIK3C2G, PIK3CD) (PIK3C2G, PIK3C2B) (PIK3C2G, PIK3C3) (PI4KB, PIK3C2A) (PI4KB, PIK3CD) (PI4KB, PIK3C2B) (PI4KB, PIK3C3) (PIK3C2A, PIK3CD) (PIK3C2A, PIK3C2B) (PIK3C2A, PIK3C3) (PIK3CD, PIK3C2B) (PIK3CD, PIK3C3) (PIK3C2B, PIK3C3) |

The result of the computation of similarity for these couples provides one of the six S distribution.

Here we compute a BP threshold for Wang's similarity measure. We use an in house implementation of this measure, available at `http://bettembourg.fr/labo/go2sip/` and illustrated by Figure 1

We repeat this process for each family to obtain six S distributions of similarity values.

**GENES TO COMPARE**

| Entrez Gene ID | Ensembl Gene ID | Ensembl Transcript ID | Uniprot ID | HGNC Symbol | Taxon | Annotation | | |
|---|---|---|---|---|---|---|---|---|
| 5286 | | | | PIK3C2A | 9606 | BP | MF | CC |
| 5287 | | | | PIK3C2B | 9606 | BP | MF | CC |
| 5288 | | | | PIK3C2G | 9606 | BP | MF | CC |
| 5289 | | | | PIK3C3 | 9606 | BP | MF | CC |
| 5290 | | | | PIK3CA | 9606 | BP | MF | CC |
| 5291 | | | | PIK3CB | 9606 | BP | MF | CC |
| 5293 | | | | PIK3CD | 9606 | BP | MF | CC |
| 5294 | | | | PIK3CG | 9606 | BP | MF | CC |
| 5297 | | | | PI4KA | 9606 | BP | MF | CC |
| 5298 | | | | PI4KB | 9606 | BP | MF | CC |

Edit the gene list    Compute Sim and Par

**SIMILARITY AND SPECIFICIY MEASURES**

Semantic similarity measure: Wang    Particularity informativeness: WangSvalue

| Biological Process | | | PI4KA (9606) | PIK3C2B (9606) | PIK3CB (9606) | PIK3CD (9606) | PI4KB (9606) | PIK3C2A (9606) | PIK3C3 (9606) | PIK3C2G (9606) | PIK3CG (9606) | PIK3CA (9606) |
|---|---|---|---|---|---|---|---|---|---|---|---|---|
| | | # Annot | 8 | 11 | 35 | 38 | 8 | 15 | 22 | 10 | 39 | 34 |
| SIM | PI4KA (9606) | 8 | 1.0 | 0.912 | 0.516 | 0.455 | 0.849 | 0.653 | 0.57 | 0.845 | 0.424 | 0.498 |
| | PIK3C2B (9606) | 11 | 0.912 | 1.0 | 0.545 | 0.475 | 0.883 | 0.688 | 0.602 | 0.895 | 0.444 | 0.534 |
| | PIK3CB (9606) | 35 | 0.516 | 0.545 | 1.0 | 0.685 | 0.521 | 0.582 | 0.552 | 0.566 | 0.658 | 0.736 |
| | PIK3CD (9606) | 38 | 0.455 | 0.475 | 0.685 | 1.0 | 0.442 | 0.501 | 0.503 | 0.5 | 0.703 | 0.676 |
| | PI4KB (9606) | 8 | 0.849 | 0.883 | 0.521 | 0.442 | 1.0 | 0.743 | 0.581 | 0.818 | 0.456 | 0.485 |
| | PIK3C2A (9606) | 15 | 0.653 | 0.688 | 0.582 | 0.501 | 0.743 | 1.0 | 0.633 | 0.636 | 0.493 | 0.56 |
| | PIK3C3 (9606) | 22 | 0.57 | 0.602 | 0.552 | 0.503 | 0.581 | 0.633 | 1.0 | 0.576 | 0.437 | 0.531 |
| | PIK3C2G (9606) | 10 | 0.845 | 0.895 | 0.566 | 0.5 | 0.818 | 0.636 | 0.576 | 1.0 | 0.461 | 0.49 |
| | PIK3CG (9606) | 39 | 0.424 | 0.444 | 0.658 | 0.703 | 0.456 | 0.493 | 0.437 | 0.461 | 1.0 | 0.637 |
| | PIK3CA (9606) | 34 | 0.498 | 0.534 | 0.736 | 0.676 | 0.485 | 0.56 | 0.531 | 0.49 | 0.637 | 1.0 |

Figure 1: **Intra-group similarity results for phosphatidylinositol kinase (pthr10048).**

# Inter-group similarity

At this step, we compute the similarities between each combination of a gene from a first group and a gene from a second group. With our six groups, we will obtain 15 inter-groups N distributions as presented by Table 3.

Table 3: **15 inter-groups N distributions**

| 15 inter-group N distributions |
|---|
| neurotransmitter gated ion channel vs histone h1/h5 |
| neurotransmitter gated ion channel vs sulfate transporter |
| neurotransmitter gated ion channel vs g-protein coupled receptor |
| neurotransmitter gated ion channel vs tyrosine-protein kinase receptor |
| neurotransmitter gated ion channel vs phosphatidylinositol kinase |
| histone h1/h5 vs sulfate transporter |
| histone h1/h5 vs g-protein coupled receptor |
| histone h1/h5 vs tyrosine-protein kinase receptor |
| histone h1/h5 vs phosphatidylinositol kinase |
| sulfate transporter vs g-protein coupled receptor |
| sulfate transporter vs tyrosine-protein kinase receptor |
| sulfate transporter vs phosphatidylinositol kinase |
| g-protein coupled receptor vs tyrosine-protein kinase receptor |
| g-protein coupled receptor vs phosphatidylinositol kinase |
| tyrosine-protein kinase receptor vs phosphatidylinositol kinase |

We have to compute the similarity value of each gene of the first group vs each gene of the second group.

For example, to obtain the N distribution comparing sulfate transporter vs phosphatidylinositol kinase, we have to compute all the pairwise similarities given in Table 4.

Table 4: **Couple of genes for which we have to compute pairwise similarity for sulfate transporter vs phosphatidylinositol kinase families**

| sulfate transporter vs phosphatidylinositol kinase inter-group couple of genes |
|---|
| (PI4KA, SLC26A7) (PI4KA, SLC26A5) (PI4KA, SLC26A4) (PI4KA, SLC26A1) (PI4KA, SLC26A2) (PI4KA, SLC26A11) (PI4KA, SLC26A8) (PI4KA, SLC26A9) (PI4KA, SLC26A3) (PI4KA, SLC26A10) (PI4KA, SLC26A6) (PIK3CB, SLC26A7) (PIK3CB, SLC26A5) (PIK3CB, SLC26A4) (PIK3CB, SLC26A1) (PIK3CB, SLC26A2) (PIK3CB, SLC26A11) (PIK3CB, SLC26A8) (PIK3CB, SLC26A9) (PIK3CB, SLC26A3) (PIK3CB, SLC26A10) (PIK3CB, SLC26A6) (PIK3CA, SLC26A7) (PIK3CA, SLC26A5) (PIK3CA, SLC26A4) (PIK3CA, SLC26A1) (PIK3CA, SLC26A2) (PIK3CA, SLC26A11) (PIK3CA, SLC26A8) (PIK3CA, SLC26A9) (PIK3CA, SLC26A3) (PIK3CA, SLC26A10) (PIK3CA, SLC26A6) (PIK3CG, SLC26A7) (PIK3CG, SLC26A5) (PIK3CG, SLC26A4) (PIK3CG, SLC26A1) (PIK3CG, SLC26A2) (PIK3CG, SLC26A11) (PIK3CG, SLC26A8) (PIK3CG, SLC26A9) (PIK3CG, SLC26A3) (PIK3CG, SLC26A10) (PIK3CG, SLC26A6) (PIK3C2G, SLC26A7) (PIK3C2G, SLC26A5) (PIK3C2G, SLC26A4) (PIK3C2G, SLC26A1) (PIK3C2G, SLC26A2) (PIK3C2G, SLC26A11) (PIK3C2G, SLC26A8) (PIK3C2G, SLC26A9) (PIK3C2G, SLC26A3) (PIK3C2G, SLC26A10) (PIK3C2G, SLC26A6) (PI4KB, SLC26A7) (PI4KB, SLC26A5) (PI4KB, SLC26A4) (PI4KB, SLC26A1) (PI4KB, SLC26A2) (PI4KB, SLC26A11) (PI4KB, SLC26A8) (PI4KB, SLC26A9) (PI4KB, SLC26A3) (PI4KB, SLC26A10) (PI4KB, SLC26A6) (PIK3C2A, SLC26A7) (PIK3C2A, SLC26A5) (PIK3C2A, SLC26A4) (PIK3C2A, SLC26A1) (PIK3C2A, SLC26A2) (PIK3C2A, SLC26A11) (PIK3C2A, SLC26A8) (PIK3C2A, SLC26A9) (PIK3C2A, SLC26A3) (PIK3C2A, SLC26A10) (PIK3C2A, SLC26A6) (PIK3CD, SLC26A7) (PIK3CD, SLC26A5) (PIK3CD, SLC26A4) (PIK3CD, SLC26A1) (PIK3CD, SLC26A2) (PIK3CD, SLC26A11) (PIK3CD, SLC26A8) (PIK3CD, SLC26A9) (PIK3CD, SLC26A3) (PIK3CD, SLC26A10) (PIK3CD, SLC26A6) (PIK3C2B, SLC26A7) (PIK3C2B, SLC26A5) (PIK3C2B, SLC26A4) (PIK3C2B, SLC26A1) (PIK3C2B, SLC26A2) (PIK3C2B, SLC26A11) (PIK3C2B, SLC26A8) (PIK3C2B, SLC26A9) (PIK3C2B, SLC26A3) (PIK3C2B, SLC26A10) (PIK3C2B, SLC26A6) (PIK3C3, SLC26A7) (PIK3C3, SLC26A5) (PIK3C3, SLC26A4) (PIK3C3, SLC26A1) (PIK3C3, SLC26A2) (PIK3C3, SLC26A11) (PIK3C3, SLC26A8) (PIK3C3, SLC26A9) (PIK3C3, SLC26A3) (PIK3C3, SLC26A10) (PIK3C3, SLC26A6) |

The result of the computation of similarity for these couples provides one of the 15 N distributions.

We use our in house implementation of Wang's similarity measure to compute all 15 N distributions.

## S and N distribution study

We can now compile all the S and N distribution in a file structured like shown in Figure 2 in order to display the distributions as boxplots and to compute Welch's t-tests to ensure the results are significant.

```
"gene1"     "gene2"     "comparison"                          "value"    "intraInter"
"PI4KA"     "PIK3C2B"   "a_[1] phosphatidylinositol kinase"    0.912     "a_Intra-group"
"PI4KA"     "PIK3CB"    "a_[1] phosphatidylinositol kinase"    0.516     "a_Intra-group"
...
"SLC26A8"   "SLC26A1 "  "a_[2] sulfate transporter"            0.542     "a_Intra-group"
...
"PI4KA"     "SLC26A8"   "b_[1] vs [2]"                         0.246     "b_Inter-group"
...
```

Figure 2: **S and N semantic similarity measure results compilation.**

The R script `boxplot.R` displayed next page draws the boxplots for a visual analysis of the distributions and computes the Welch's t-tests. If Welch's t-test cannot highlight a significant difference between S and N distributions, we have to chose other groups of genes and restart all the process. With the data used, the boxplot obtained is displayed in the part A of the Figure 8 of our paper "Interpretation of semantic similarity and particularity for functional analysis of gene set" and the result of the Welch's t-tests are in Supporting Information file S2. These Welch's t-test results allow to consider the S and N distribution significantly different.

```r
bxthreshold <- read.table("/data/similarity_BP.csv",
                          sep="\t",
                          header=TRUE)
comparison <- bxthreshold$comparison
similarity <- bxthreshold$value
intraInter <- bxthreshold$intraInter
mat=matrix(c(1,1,1,2,2,2),nrow=1)
layout(mat)
boxplot(similarity ~ comparison,
        data = bxthreshold,
        subset = intraInter == "a_Intra-group",
        col = "lightblue",
        xlab = "groups of genes",
        ylab = "Distribution of the semantic similarity measured",
        boxwex = 0.75,
        ylim = c(0, 1.1),
        las = 2)
boxplot(similarity ~ comparison, data = bxthreshold,
        add = TRUE,
        subset = intraInter == "b_Inter-group",
        xaxt = "n",
        yaxt = "n",
        col = "yellow",
        boxwex = 0.75,
        ylim = c(0, 1.1),
        las = 2)
boxplot(similarity ~ intraInter,
        data = bxthreshold,
        subset = intraInter == "a_Intra-group",
        col = "lightblue",
        ylab = "Distribution of the semantic similarity measured",
        ylim = c(0, 1.1))
boxplot(similarity ~ intraInter,
        data = bxthreshold,
        add = TRUE,
        subset = intraInter == "b_Inter-group",
        xaxt = "n",
        yaxt = "n",
        col = "yellow",
        ylim = c(0, 1.1))

sink("/tests/welch_BP.txt")
cat("Comparison of the boxes Intra-groups and Inter-groups")
tst <- t.test(similarity ~ intraInter, data = bxthreshold)
print(tst)
for (i in unique(comparison)){
  for (j in unique(comparison)){
    if (i != j){
      t1 <- subset(similarity, (comparison == i))
      t2 <- subset(similarity, (comparison == j))
      cat("==================================================\n")
      cat("Comparison of the boxes [", i, "] and [", j, "]")
      tst <- t.test(t1, t2)
      print(tst)
    }
  }
}
sink()
```

boxplots.R

# Step 3: Overlap study

If the S and N distributions have no overlap between the ranges (min, max), we define the threshold $\tau_{sim}$ using any value between $\tau_S$ (the lowest value of S) and $\tau_N$ (the highest value of N). Else, like with the data we used here, there are some false negatives (FN) and some false positives (FP).

The lowest value of all S distributions is $\tau_N$, the threshold value under which the two compared genes are non-similar. There cannot be any FN below $\tau_N$, but there will be some FPs above $\tau_N$ as S and N distributions overlap. The highest value of all N distributions is $\tau_S$, the threshold value above which the two compared genes are similar. There cannot be any FP above $\tau_S$, but there will be some FNs below $\tau_S$ as S and N distributions overlap.

To find the best threshold of similarity $\tau_{sim}$, we have to study the variation of the proportions of FPs and FNs when varying $\tau_{sim}$ between $\tau_N$ and $\tau_S$. The Python script `thresholdVariation.py` computes these FP and FN proportions and allows to find the minimum the sum of these FP and FN proportions as showed in Figure 3.

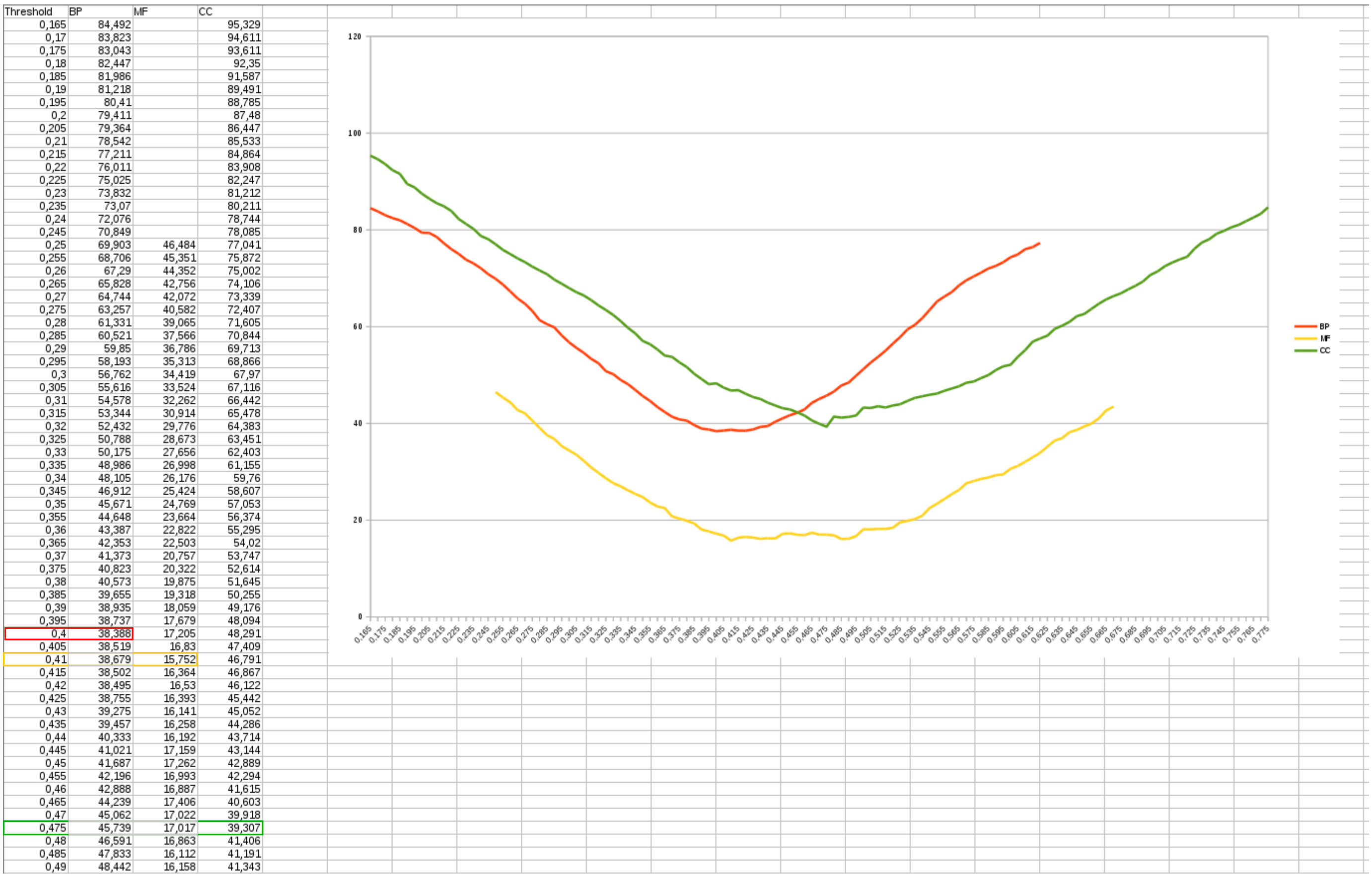

| Threshold | BP | MF | CC |
|---|---|---|---|
| 0,165 | 84,492 | | 95,329 |
| 0,17 | 83,823 | | 94,611 |
| 0,175 | 83,043 | | 93,611 |
| 0,18 | 82,447 | | 92,35 |
| 0,185 | 81,986 | | 91,587 |
| 0,19 | 81,218 | | 89,491 |
| 0,195 | 80,41 | | 88,785 |
| 0,2 | 79,411 | | 87,48 |
| 0,205 | 79,364 | | 86,447 |
| 0,21 | 78,542 | | 85,533 |
| 0,215 | 77,211 | | 84,864 |
| 0,22 | 76,011 | | 83,908 |
| 0,225 | 75,025 | | 82,247 |
| 0,23 | 73,832 | | 81,212 |
| 0,235 | 73,07 | | 80,211 |
| 0,24 | 72,076 | | 78,744 |
| 0,245 | 70,849 | | 78,085 |
| 0,25 | 69,903 | 46,484 | 77,041 |
| 0,255 | 68,706 | 45,351 | 75,872 |
| 0,26 | 67,29 | 44,352 | 75,002 |
| 0,265 | 65,828 | 42,756 | 74,106 |
| 0,27 | 64,744 | 42,072 | 73,339 |
| 0,275 | 63,257 | 40,582 | 72,407 |
| 0,28 | 61,331 | 39,065 | 71,605 |
| 0,285 | 60,521 | 37,566 | 70,844 |
| 0,29 | 59,85 | 36,786 | 69,713 |
| 0,295 | 58,193 | 35,313 | 68,866 |
| 0,3 | 56,762 | 34,419 | 67,97 |
| 0,305 | 55,616 | 33,524 | 67,116 |
| 0,31 | 54,578 | 32,262 | 66,442 |
| 0,315 | 53,344 | 30,914 | 65,478 |
| 0,32 | 52,432 | 29,776 | 64,383 |
| 0,325 | 50,788 | 28,673 | 63,451 |
| 0,33 | 50,175 | 27,656 | 62,403 |
| 0,335 | 48,986 | 26,998 | 61,155 |
| 0,34 | 48,105 | 26,176 | 59,76 |
| 0,345 | 46,912 | 25,424 | 58,607 |
| 0,35 | 45,671 | 24,769 | 57,053 |
| 0,355 | 44,648 | 23,664 | 56,374 |
| 0,36 | 43,387 | 22,822 | 55,295 |
| 0,365 | 42,353 | 22,503 | 54,02 |
| 0,37 | 41,373 | 20,757 | 53,747 |
| 0,375 | 40,823 | 20,322 | 52,614 |
| 0,38 | 40,573 | 19,875 | 51,645 |
| 0,385 | 39,655 | 19,318 | 50,255 |
| 0,39 | 38,935 | 18,059 | 49,176 |
| 0,395 | 38,737 | 17,679 | 48,094 |
| 0,4 | 38,388 | 17,205 | 48,291 |
| 0,405 | 38,519 | 16,83 | 47,409 |
| 0,41 | 38,679 | 15,752 | 46,791 |
| 0,415 | 38,502 | 16,364 | 46,867 |
| 0,42 | 38,495 | 16,53 | 46,122 |
| 0,425 | 38,755 | 16,393 | 45,442 |
| 0,43 | 39,275 | 16,141 | 45,052 |
| 0,435 | 39,457 | 16,258 | 44,286 |
| 0,44 | 40,333 | 16,192 | 43,714 |
| 0,445 | 41,021 | 17,159 | 43,144 |
| 0,45 | 41,687 | 17,262 | 42,889 |
| 0,455 | 42,196 | 16,993 | 42,294 |
| 0,46 | 42,888 | 16,887 | 41,615 |
| 0,465 | 44,239 | 17,406 | 40,603 |
| 0,47 | 45,062 | 17,022 | 39,918 |
| 0,475 | 45,739 | 17,017 | 39,307 |
| 0,48 | 46,591 | 16,863 | 41,406 |
| 0,485 | 47,833 | 16,112 | 41,191 |
| 0,49 | 48,442 | 16,158 | 41,343 |



Figure 3: **Sum of FP and FN proportions when varying $\tau_{sim}$ between $\tau_N$ and $\tau_S$.**

```python
#! /usr/bin/env python
# -*- coding: utf-8 -*-

"""
gene2group is a dictionary with genes as keys and a group
name as value. Example:
gene2group = {"PI4KA":"phosphatidylinositol kinase",
              "PIK3C2B":"phosphatidylinositol kinase",
              "PIK3CB":"phosphatidylinositol kinase",
              "SLC26A8":"sulfate transporter",
              "SLC26A1":"sulfate transporter",
              "SLC26A4":"sulfate transporter"}

similarity is a dictionary with genes as first and second
level keys and a similarity value as keys. Example:
similarity = {"PI4KA":{"PIK3C2B":0.912,
                       "PIK3CB":0.516,
                       "SLC26A8":0.246,
                       "SLC26A1":0.35,
                       "SLC26A4":0.244},
              "PIK3C2B":{"PIK3CB":0.545,
                         "SLC26A8":0.237,
                         "SLC26A1":0.352,
                         "SLC26A4":0.228},
              "PIK3CB":{"SLC26A8":0.255,
                        "SLC26A1":0.316,
                        "SLC26A4":0.257},
              "SLC26A8":{"SLC26A1":0.542,
                         "SLC26A4":0.555},
              "SLC26A1":{"SLC26A4":0.543}}
"""

intra = []
inter = []
tauN = 164 # (tau N * 1000)
tauS = 618 # (tau S * 1000)

for gene1 in similarity.keys():
    for gene2 in similarity[gene1]:
        if gene2group[gene1] == gene2group[gene2]:
            intra.append(similarity[gene1][gene2])
        else:
            inter.append(similarity[gene1][gene2])

for thr in range(tauN, tauS, 5):
    fn = 0
    fp = 0
    for value in intra:
        if value <= thr/1000.0:
            fn+=1
    for value in inter:
        if value >= thr/1000.0:
            fp += 1
    fnProportion = round(fn/float(len(intra))*100, 3)
    fpProportion = round(fp/float(len(inter))*100, 3)
    print thr/1000.0, fnProportion + fpProportion
```

thresholdVariation.py
