# Supplementary material for: Optimal Threshold Determination for Interpreting Semantic Similarity and Particularity: Application to the Comparison of Gene Sets and Metabolic Pathways Using GO and ChEBI
Source: PLoS One. 2015 Jul 31;10(7):e0133579. doi: 10.1371/journal.pone.0133579 (PMC4521860; doi:10.1371/journal.pone.0133579)
Supplement: S6 File — (PDF) [file pone.0133579.s006.pdf]

Particularity threshold computation: supplementary figures and tables

Charles Bettembourg, Christian Diot, Olivier Dameron

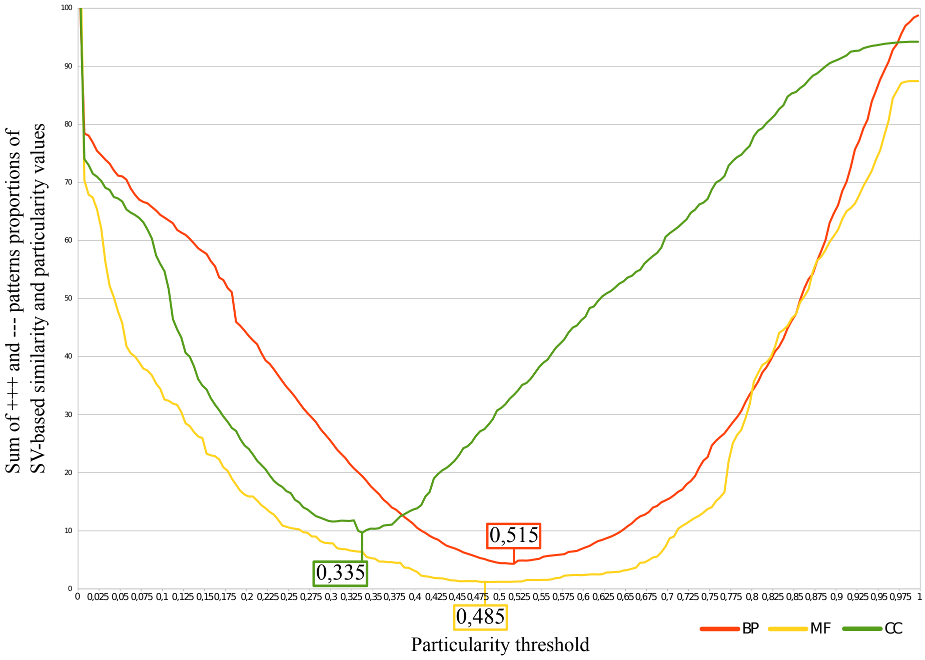

Figure 1: **Determination of the SV-based particularity threshold.** The minimum of “+ + +” and “- - -” pattern proportions gives the particularity threshold.

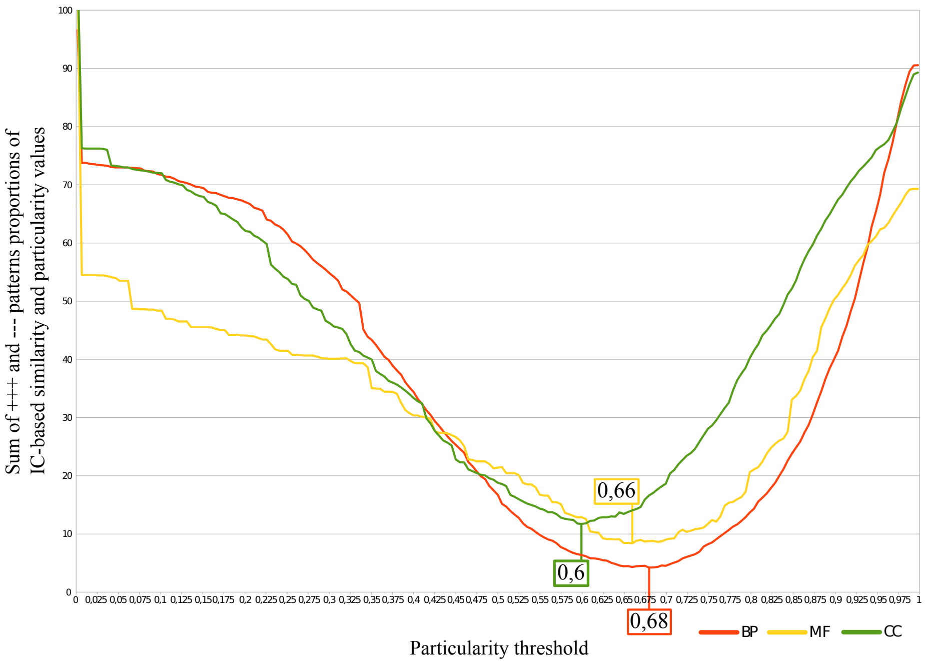

Figure 2: **Determination of the IC-based particularity threshold.** The minimum of "+ + +" and "- - -" pattern proportions gives the particularity threshold.

Table 1: **Particularity threshold variations considering full and partial datasets (SV-based measure)**

Set	Par. threshold	+ + + (%)	- - - (%)
BP set	0.515	2.014	2.303
BP set w/o histone	0.495	1.974	2.506
BP set w/o g-protein... receptor	0.515	2.129	2.29
BP set w/o neurotr... channel	0.515	1.715	2.764
BP set w/o tyrosine-... receptor	0.49	1.515	1.648
BP set w/o phosphat...-kinase	0.51	2.154	2.1
BP set w/o sulfate transporter	0.495	1.632	2.624
MF set	0.485	0.894	0.301
MF set w/o histone	0.465	1.121	0.232
MF set w/o g-protein... receptor	0.45	1.795	0.221
MF set w/o neurotr... channel	0.35	0.857	0.599
MF set w/o tyrosine-... receptor	0.485	0.301	0.399
MF set w/o phosphat...-kinase	0.485	1.013	0.253
MF set w/o sulfate transporter	0.485	0.946	0.245
CC set	0.335	5.013	4.677
CC set w/o Chr... maintenance	0.335	5.04	4.583
CC set w/o Mitoch... import	0.335	5.427	3.921
CC set w/o Potassium channels	0.28	7.15	4.902
CC set w/o Protein folding	0.335	4.218	4.97
CC set w/o Term... biosynthesis	0.355	3.873	4.086

This table summarizes the particularity thresholds obtained considering each complete dataset or all the groups of a dataset except the SV-based particularity measure. The numbers given for “+ + +” and “- - -” are the proportions of non informative cases that the threshold admits in the comparison results.

Table 2: **Particularity threshold variations considering full and partial datasets (IC-based measure)**

Set	Par. threshold	+ + + (%)	- - - (%)
BP set	0.68	1.598	2.665
BP set w/o histone	0.68	1.671	2.896
BP set w/o g-protein... receptor	0.685	1.279	1.71
BP set w/o neurotr... channel	0.69	1.619	1.823
BP set w/o tyrosine-... receptor	0.68	2.168	1.98
BP set w/o phosphat...-kinase	0.685	1.77	2.279
BP set w/o sulfate transporter	0.68	1.484	3.048
MF set	0.66	2.935	5.506
MF set w/o histone	0.66	3.117	5.957
MF set w/o g-protein... receptor	0.66	0.569	1.275
MF set w/o neurotr... channel	0.69	2.98	10.424
MF set w/o tyrosine-... receptor	0.74	1.463	5.248
MF set w/o phosphat...-kinase	0.66	3.015	5.723
MF set w/o sulfate transporter	0.65	2.478	5.956
CC set	0.6	7.155	4.622
CC set w/o Chr... maintenance	0.6	8.717	4.87
CC set w/o Mitoch... import	0.605	7.168	3.983
CC set w/o Potassium channels	0.56	8.427	4.338
CC set w/o Protein folding	0.595	8.026	3.039
CC set w/o Term... biosynthesis	0.6	5.96	4.573

This table summarizes the particularity thresholds obtained considering each complete dataset or all the groups of a dataset except the IC-based particularity measure. The numbers given for "+ + +" and "- - -" are the proportions of non-informative cases that the threshold admits in the comparison results.
