# Supplementary material for: Optimal Threshold Determination for Interpreting Semantic Similarity and Particularity: Application to the Comparison of Gene Sets and Metabolic Pathways Using GO and ChEBI
Source: PLoS One. 2015 Jul 31;10(7):e0133579. doi: 10.1371/journal.pone.0133579 (PMC4521860; doi:10.1371/journal.pone.0133579)
Supplement: S8 File — (PDF) [file pone.0133579.s008.pdf]

Similarity and particularity values of PPARα, PPARβ and PPARγ between six species

Charles Bettembourg, Christian Diot, Olivier Dameron

Table 1: **SV-based BP similarity and particularity measured between orthologs and paralogs of the PPAR family.**

BP			α mmu	α rno	α mamu	α hsa	α cca	α bta	β mmu	β rno	β mamu	β hsa	β cca	β bta	γ mmu	γ rno	γ mamu	γ hsa	γ cca	γ bta
		#Annot	32	30	20	39	21	21	30	49	25	37	25	25	61	56	38	65	38	38
SIM	α mmu	32	1	0.983	0.847	0.946	0.859	0.859	0.608	0.601	0.611	0.628	0.611	0.611	0.642	0.654	0.616	0.65	0.616	0.616
	α rno	30		1	0.869	0.945	0.877	0.877	0.598	0.592	0.6	0.598	0.6	0.6	0.645	0.657	0.628	0.644	0.628	0.628
	α mamu	20			1	0.814	0.993	0.993	0.625	0.563	0.634	0.612	0.634	0.634	0.606	0.614	0.627	0.601	0.627	0.627
	α hsa	39				1	0.831	0.831	0.634	0.623	0.637	0.655	0.637	0.637	0.663	0.677	0.634	0.694	0.634	0.634
	α cca	21					1	1	0.63	0.578	0.64	0.626	0.64	0.64	0.612	0.625	0.621	0.608	0.621	0.621
	α bta	21						1	0.63	0.578	0.64	0.626	0.64	0.64	0.612	0.625	0.621	0.608	0.621	0.621
	β mmu	30							1	0.83	0.948	0.917	0.948	0.948	0.647	0.643	0.663	0.638	0.663	0.663
	β rno	49								1	0.823	0.822	0.823	0.823	0.715	0.714	0.644	0.711	0.644	0.644
	β mamu	25									1	0.929	1	1	0.642	0.65	0.68	0.644	0.68	0.68
	β hsa	37										1	0.929	0.929	0.642	0.652	0.662	0.661	0.662	0.662
	β cca	25											1	1	0.642	0.65	0.68	0.644	0.68	0.68
	β bta	25												1	0.642	0.65	0.68	0.644	0.68	0.68
	γ mmu	61													1	0.978	0.882	0.959	0.882	0.882
	γ rno	56														1	0.896	0.97	0.896	0.896
	γ mamu	38															1	0.868	1	1
	γ hsa	65																1	0.868	0.868
	γ cca	38																	1	1
	γ bta	38																		1
PAR	α mmu	32	0	0	0.112	0	0.112	0.112	0.481	0.442	0.487	0.474	0.487	0.487	0.312	0.315	0.437	0.308	0.437	0.437
	α rno	30	0	0	0.112	0	0.112	0.112	0.481	0.442	0.487	0.474	0.487	0.487	0.312	0.315	0.437	0.308	0.437	0.437
	α mamu	20	0	0	0	0	0	0	0.424	0.408	0.429	0.414	0.429	0.429	0.282	0.285	0.405	0.277	0.405	0.405
	α hsa	39	0.167	0.167	0.259	0	0.259	0.259	0.502	0.469	0.507	0.483	0.507	0.507	0.398	0.4	0.502	0.362	0.502	0.502
	α cca	21	0	0	0	0	0	0	0.424	0.408	0.429	0.414	0.429	0.429	0.282	0.285	0.405	0.277	0.405	0.405
	α bta	21	0	0	0	0	0	0	0.424	0.408	0.429	0.414	0.429	0.429	0.282	0.285	0.405	0.277	0.405	0.405
	β mmu	30	0.441	0.441	0.449	0.357	0.449	0.449	0	0.009	0.144	0.127	0.144	0.144	0.265	0.364	0.412	0.361	0.412	0.412
	β rno	49	0.603	0.603	0.626	0.548	0.626	0.626	0.346	0	0.435	0.405	0.435	0.435	0.424	0.491	0.578	0.489	0.578	0.578
	β mamu	25	0.355	0.355	0.362	0.256	0.362	0.362	0	0	0	0	0	0	0.27	0.27	0.327	0.27	0.327	0.327
	β hsa	37	0.417	0.417	0.423	0.313	0.423	0.423	0.101	0.073	0.119	0	0.119	0.119	0.341	0.341	0.391	0.325	0.391	0.391
	β cca	25	0.355	0.355	0.362	0.256	0.362	0.362	0	0	0	0	0	0	0.27	0.27	0.327	0.27	0.327	0.327
	β bta	25	0.355	0.355	0.362	0.256	0.362	0.362	0	0	0	0	0	0	0.27	0.27	0.327	0.27	0.327	0.327
	γ mmu	61	0.548	0.548	0.581	0.526	0.581	0.581	0.551	0.467	0.619	0.61	0.619	0.619	0	0.104	0.32	0.102	0.32	0.32
	γ rno	56	0.498	0.498	0.534	0.473	0.534	0.534	0.567	0.475	0.575	0.565	0.575	0.575	0	0	0.241	0	0.241	0.241
	γ mamu	38	0.456	0.456	0.489	0.422	0.489	0.489	0.473	0.427	0.483	0.47	0.483	0.483	0	0	0	0	0	0
	γ hsa	65	0.52	0.52	0.554	0.469	0.554	0.554	0.588	0.501	0.597	0.577	0.597	0.597	0.051	0.053	0.282	0	0.282	0.282
	γ cca	38	0.456	0.456	0.489	0.422	0.489	0.489	0.473	0.427	0.483	0.47	0.483	0.483	0	0	0	0	0	0
	γ bta	38	0.456	0.456	0.489	0.422	0.489	0.489	0.473	0.427	0.483	0.47	0.483	0.483	0	0	0	0	0	0

Green cells contain similarity values greater than τ_s, red cells contain similarity values lower than τ_s, yellow cells contain values greater than τ_{par} and blue cells contain values lower than τ_{par}. All orthologs have a "+ - -" pattern and some paralogs have a "- + -" or a "+ + -" pattern. The number of annotations for each ortholog is given by the "#Annot" line and column.

Table 2: **SV-based MF similarity and particularity measured between orthologs and paralogs of the PPAR family.**

BP			α mmu	α rno	α mamu	α hsa	α cca	α bta	β mmu	β rno	β mamu	β hsa	β cca	β bta	γ mmu	γ rno	γ mamu	γ hsa	γ cca	γ bta
		#Annot	32	30	20	39	21	21	30	49	25	37	25	25	61	56	38	65	38	38
SIM	α mmu	32	1	0.983	0.847	0.946	0.859	0.859	0.608	0.601	0.611	0.628	0.611	0.611	0.642	0.654	0.616	0.65	0.616	0.616
	α rno	30		1	0.869	0.945	0.877	0.877	0.598	0.592	0.6	0.598	0.6	0.6	0.645	0.657	0.628	0.644	0.628	0.628
	α mamu	20			1	0.814	0.993	0.993	0.625	0.563	0.634	0.612	0.634	0.634	0.606	0.614	0.627	0.601	0.627	0.627
	α hsa	39				1	0.831	0.831	0.634	0.623	0.637	0.655	0.637	0.637	0.663	0.677	0.634	0.694	0.634	0.634
	α cca	21					1	1	0.63	0.578	0.64	0.626	0.64	0.64	0.612	0.625	0.621	0.608	0.621	0.621
	α bta	21						1	0.63	0.578	0.64	0.626	0.64	0.64	0.612	0.625	0.621	0.608	0.621	0.621
	β mmu	30							1	0.83	0.948	0.917	0.948	0.948	0.647	0.643	0.663	0.638	0.663	0.663
	β rno	49								1	0.823	0.822	0.823	0.823	0.715	0.714	0.644	0.711	0.644	0.644
	β mamu	25									1	0.929	1	1	0.642	0.65	0.68	0.644	0.68	0.68
	β hsa	37										1	0.929	0.929	0.642	0.652	0.662	0.661	0.662	0.662
	β cca	25											1	1	0.642	0.65	0.68	0.644	0.68	0.68
	β bta	25												1	0.642	0.65	0.68	0.644	0.68	0.68
	γ mmu	61													1	0.978	0.882	0.959	0.882	0.882
	γ rno	56														1	0.896	0.97	0.896	0.896
	γ mamu	38															1	0.868	1	1
	γ hsa	65																1	0.868	0.868
	γ cca	38																	1	1
	γ bta	38																		1
PAR	α mmu	32	0	0	0.112	0	0.112	0.112	0.481	0.442	0.487	0.474	0.487	0.487	0.312	0.315	0.437	0.308	0.437	0.437
	α rno	30	0	0	0.112	0	0.112	0.112	0.481	0.442	0.487	0.474	0.487	0.487	0.312	0.315	0.437	0.308	0.437	0.437
	α mamu	20	0	0	0	0	0	0	0.424	0.408	0.429	0.414	0.429	0.429	0.282	0.285	0.405	0.277	0.405	0.405
	α hsa	39	0.167	0.167	0.259	0	0.259	0.259	0.502	0.469	0.507	0.483	0.507	0.507	0.398	0.4	0.502	0.362	0.502	0.502
	α cca	21	0	0	0	0	0	0	0.424	0.408	0.429	0.414	0.429	0.429	0.282	0.285	0.405	0.277	0.405	0.405
	α bta	21	0	0	0	0	0	0	0.424	0.408	0.429	0.414	0.429	0.429	0.282	0.285	0.405	0.277	0.405	0.405
	β mmu	30	0.441	0.441	0.449	0.357	0.449	0.449	0	0.009	0.144	0.127	0.144	0.144	0.265	0.364	0.412	0.361	0.412	0.412
	β rno	49	0.603	0.603	0.626	0.548	0.626	0.626	0.346	0	0.435	0.405	0.435	0.435	0.424	0.491	0.578	0.489	0.578	0.578
	β mamu	25	0.355	0.355	0.362	0.256	0.362	0.362	0	0	0	0	0	0	0.27	0.27	0.327	0.27	0.327	0.327
	β hsa	37	0.417	0.417	0.423	0.313	0.423	0.423	0.101	0.073	0.119	0	0.119	0.119	0.341	0.341	0.391	0.325	0.391	0.391
	β cca	25	0.355	0.355	0.362	0.256	0.362	0.362	0	0	0	0	0	0	0.27	0.27	0.327	0.27	0.327	0.327
	β bta	25	0.355	0.355	0.362	0.256	0.362	0.362	0	0	0	0	0	0	0.27	0.27	0.327	0.27	0.327	0.327
	γ mmu	61	0.548	0.548	0.581	0.526	0.581	0.581	0.551	0.467	0.619	0.61	0.619	0.619	0	0.104	0.32	0.102	0.32	0.32
	γ rno	56	0.498	0.498	0.534	0.473	0.534	0.534	0.567	0.475	0.575	0.565	0.575	0.575	0	0	0.241	0	0.241	0.241
	γ mamu	38	0.456	0.456	0.489	0.422	0.489	0.489	0.473	0.427	0.483	0.47	0.483	0.483	0	0	0	0	0	0
	γ hsa	65	0.52	0.52	0.554	0.469	0.554	0.554	0.588	0.501	0.597	0.577	0.597	0.597	0.051	0.053	0.282	0	0.282	0.282
	γ cca	38	0.456	0.456	0.489	0.422	0.489	0.489	0.473	0.427	0.483	0.47	0.483	0.483	0	0	0	0	0	0
	γ bta	38	0.456	0.456	0.489	0.422	0.489	0.489	0.473	0.427	0.483	0.47	0.483	0.483	0	0	0	0	0	0

Green cells contain similarity values greater than τ_s, red cells contain similarity values lower than τ_s, yellow cells contain values greater than τ_{par} and blue cells contain values lower than τ_{par}. All orthologs have a "+ - -" pattern and some paralogs have a "+ + -" pattern. The number of annotations for each ortholog is given by the "#Annot" line and column.
